# Petrogenesis and tectonic evolution of tourmaline- bearing leucogranites, Sikait area, South Eastern Desert of Egypt utilizing mineralogical and bulk rock analysis

**DOI:** 10.1038/s41598-025-06155-x

**Published:** 2025-06-20

**Authors:** Gehad M. Saleh, Mohamed S. Kamar, Farrage M. Khaleal, Mokhles K. Azer, Taher Nasr, El Saeed R. Lasheen

**Affiliations:** 1 Nuclear Materials Authority, P. O. Box 530, El-Maadi, Cairo, Egypt; 2 National Research Centre, Cairo, Egypt; 3 Geology Department, Faculty of Science, Al-Azhar University, Cairo, Egypt

**Keywords:** Tourmaline, S-type leucogranites, Mineral chemistry, Bulk rock geochemistry, Geodynamic evolution, Geochemistry, Mineralogy, Petrology, Solid Earth sciences

## Abstract

The current research discuss in detail the tourmaline distribution in Sikait leucogranites in order to deduce its genesis and type. We conduct new detailed geological, petrographical, mineralogical, and geochemical examinations to understand the Arabian Nubian Shield development by investigation of such the examined leucogranites. Tourmaline occurs as disseminated or cluster nodular within coarse-grained leucogranites. Geochemically, the examined leucogranites have high contents of SiO_2_ (69.44–75.87 wt%), and total alkalis (mean > 7) with low mean CaO (0.4 wt%), Fe_2_O_3_ (1.93 wt%), and Mg# (14.59) values. They share features of calc-alkaline, strongly peraluminous (A/CNK > 1.1), with high contents of Zn (av. 266.68 ppm), Pb (av. 29.13 ppm), Rb/Sr (av. 22), Al_2_O_3_/TiO_2_ (av. 832.6), FeO/MgO (av. 12.24). They are remarkably enriched in semi-volatile elements (Pb = 12–235 ppm), and LILEs (Rb = 192–679 ppm) relative to HFSEs (e.g. Zr, U and Nb) with notable strong Ba, Sr and Ti negative anomalies. They are depleted in ∑REEs (av. 19.1 ppm) and reveal parallel, uniform patterns slightly notable depletion of HREEs in comparison with LREEs. They reveal extreme pronounced Eu (av. Eu/Eu*= 0.02) negative and Ce/Ce* (0.76–1.12) positive anomalies. The examined rocks have prominent tetrad effect (M-type) as indicated by Irber and Lambda methods. Based up on conventional geochemical diagrams, the examined rocks are post-collisional S-type granites derived by partial degree of the clay-rich pelite rocks melting followed by extreme fractional crystallization processes during post-collisional extension episode at temperatures (663 –786 °C) based on saturation temperature of zircon. The investigated tourmaline nodules are of alkali group and foitite end-member.

**Supplementary Information:**

The online version contains supplementary material available at 10.1038/s41598-025-06155-x.

## Introduction

 Boron (B) is a rare lithophile and easily remobilized by fluids/hydrothermal solutions^1^. B has two stable isotopes,^11^B and ^10^B, with the relative abundance of ^11^B being approximately 80% and ^10^B about 20% in nature^1,2^. B represents the main component (hydrous boron-silicate) of tourmaline minerals^1,3–6^. The resistant tourmaline is spatially distributed in a diverse environments (pressure and temperature conditions) related to magmatic/hydrothermal deposits such as highly evolved granitic and linked rocks (pegmatites)^7^ Mg-bearing schists^3^ volcanic-hosted sulphide^8^ and orogenic gold deposits^2^. B-bearing highly evolved granitic rocks/fluids intruding Al-rich sediments are reasonable for tourmaline formation^3,5,8,9^. It is widely observed that the highly evolved granitic and linked rocks (magmatic) represent that dominant source of fluids that sweep metals from rocks which are intruded^5^. Therefore, magmatic fluids are used as a key to manifest the variable processes of ore deposits mechanism. Tourmaline has a geochemical affinity of the environment from which it crystallizes, where it combine several cations with variable charge and size and hence it has a diverse members^9^. This give rise variable color with prismatic morphology, so the tourmaline members are represented a semi-precious gemstone. Tourmaline is a stable mineral that preserves chemical signatures of earlier growth periods^9,10^. Consequently, composition of tourmaline can be utilized to track for evolution of magma and source ore-forming fluids as a result of diverse in its P/T stability as well as boron isotope composition^1,2,5,7,10^. Tourmaline deposits is commonly black with prismatic as well as fibrous morphology^3,5,8,9^.

Egyptian tourmaline is restricted to Nugrus-Sikait belt^11^ and it is origin is related to hydrothermal fluids produced from interaction between felsic magma and Mg-rich rocks. Furthermore, tourmaline minerals are observed in schist (that represent a suitable host rock for tourmaline deposition) in Sikait area^3^. They conclude that it is formed by injection of B-bearing felsic magma within schist. From the other side, Abu El-Enen and Okrusch^12^ stated that the tourmaline)metamorphic-type) inclusions occur in metapelites, which is formed during retrograde and prograde metamorphism.

In order to manifest the petrogenesis, natural type, and geodynamic history of tourmaline-bearing leucogranites, as well as origin of tourmaline mineral, we prepared and integrate new field, petrology and mineral chemistry of tourmaline-bearing leucogranites in Sikait area. Additionally, the current research discuss in detail the tourmaline distribution in Sikait leucogranites in order to deduce its genesis and type.

## Geological background

The dominant Neoproterozoic rocks are crop out in Northern sector Eastern Desert (ED), of Arabian Nubian Shield and the largest East African Orogen^13–15^. Its dominated by ophiolites, accreted arc assemblages, Dokhan volcanics, and variable intrusive granitic rocks^16–23^. Each dominant rocks are distinguished by significant mineralization. The former (ophiolite certainly ultramafics) is characterized by magnesite and chromite mineralization, whereas arc assemblage are characterized by BIF, volcanogenic massive sulphide^24^. Th, Nb, Sn, Ta, W, and U distinguish the granitic rocks particularly highly evolved granitic rocks^22,25,26^ and are widely used as a decorative stone^27,28^.

**Fig. 1:**
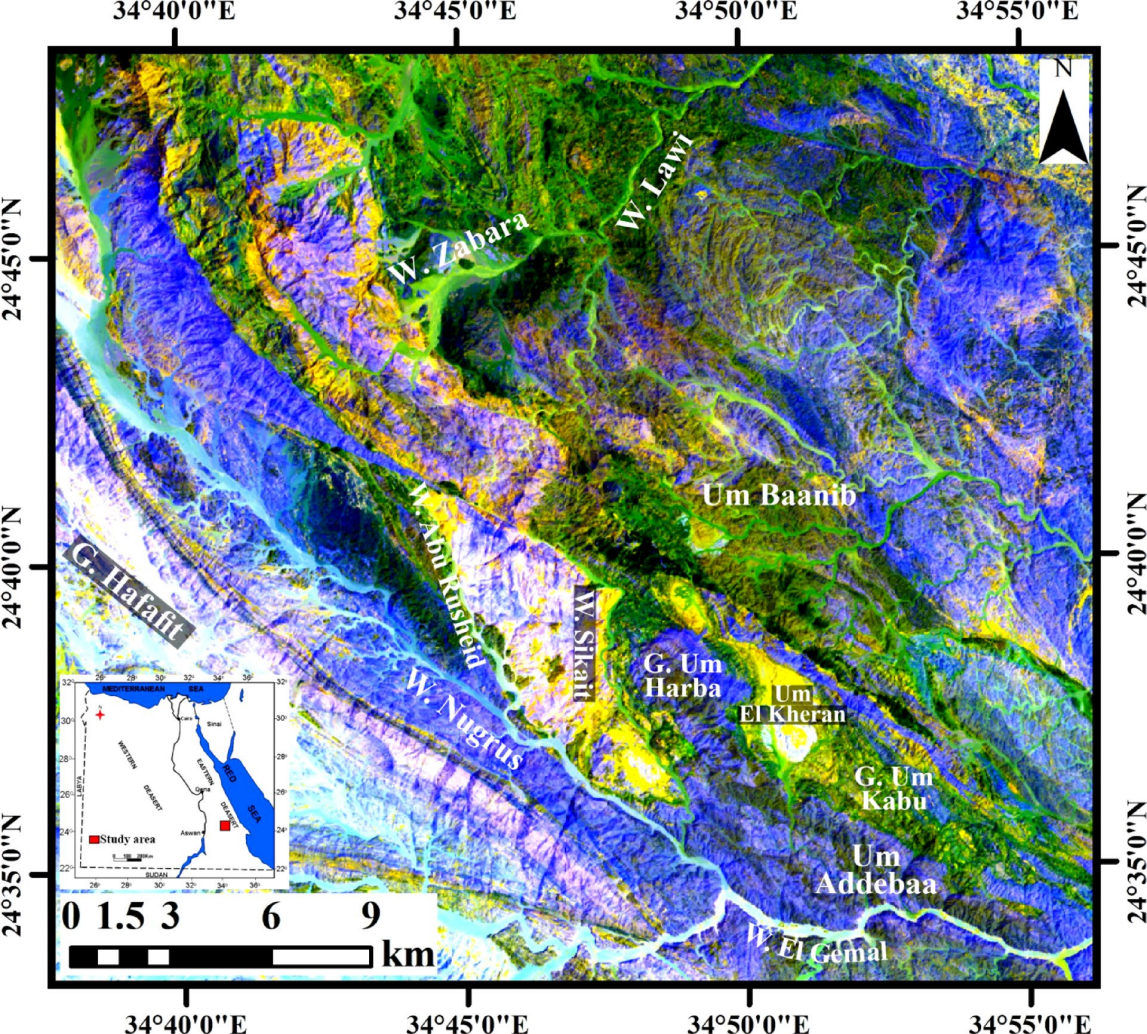
A view of Nugrus-Sikait area, southeastern, Desert, Egypt (through Arc GIS 10.4 and ENVI 5.3. with Landsat-8 image acquisition date: Sep. 8, 2021, path 173, and row 43. The Landsat-8 Source: http://earthexplorer.usgs.gov).

**Fig. 2:**
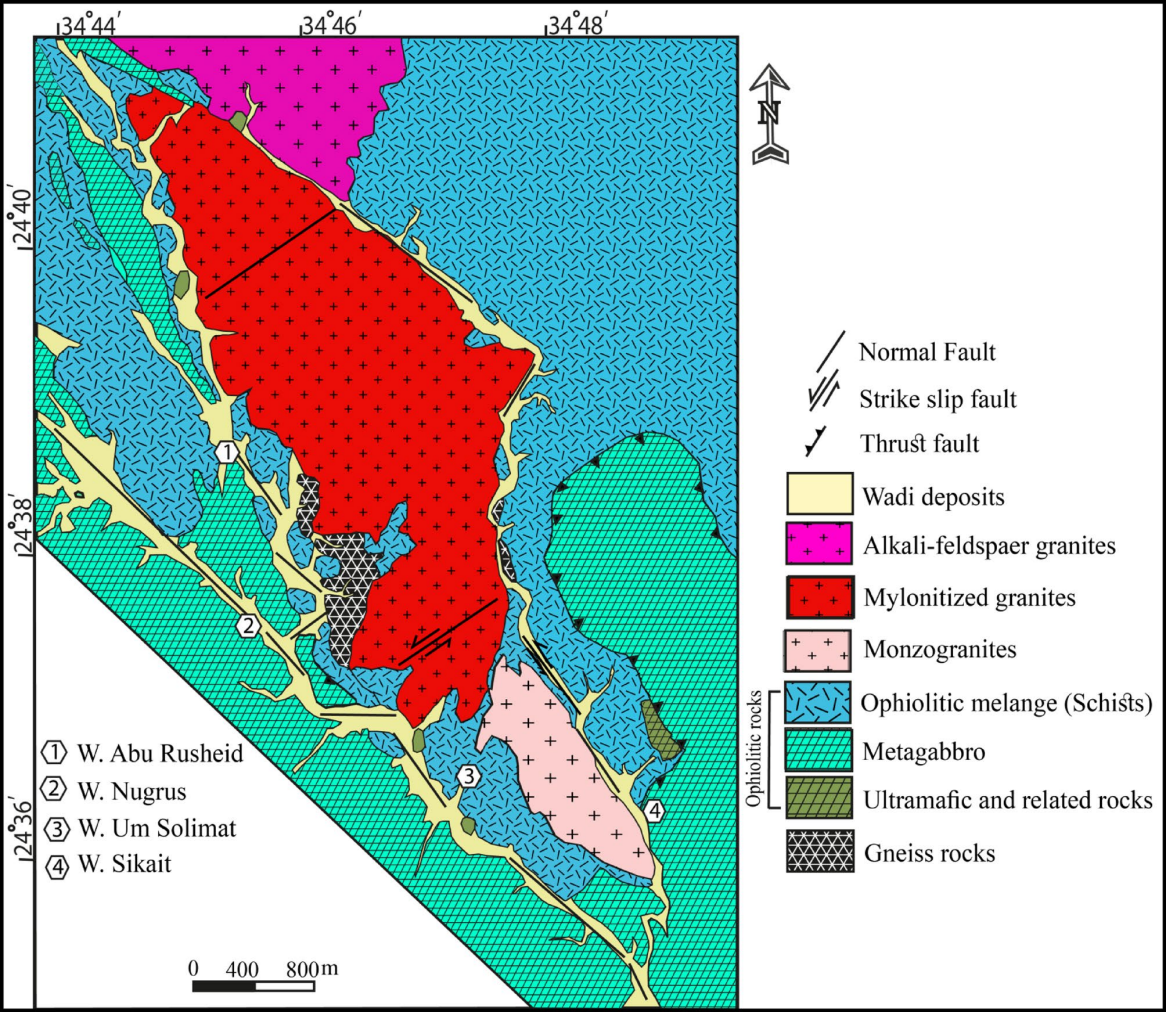
Nugrus-Sikait geologic map^31^ (by using Adobe Illustrator program CS5).

Sikait area lies in south Eastern Desert that is characterized by fish-like shape (Fig. 1)^29^. It is belong to major that fault NW-SE direction comprising Zabara, Abu Rusheid, Hafafit, Nugrus, Sikait and Um Addebaa areas^30^. Gneisses, dismembered ophiolitic rocks, and variable granitic rocks are the dominant rock units (Fig. 2). On the eastern sector of W. Abu Rusheid, gneisses are cropped out forming about 3 km^2^ and small exposure occur in W. Sikait. They follow the NW-SE structure trend of Nugrus-Sikait belt. Along W. Abu Rusheid, they are invaded by numerous lamprophyre dikes causing rare-metals enrichment^30^. Ultramafic rocks, layered metagabbro, and ophiolitic mélange (schistose rocks) are the exposed ophiolitic sequence. Small sheet of serpentinites and their alteration products (talc-carbonates) are exposed along the eastern side of W. Sikait. Layered metagabbros and mélange (including talc schist, mica schist, tourmaline-garnetiferous-biotite schist, and graphite schist are main rock units of the ophiolitic sequence^31^. They cover most sectors of the studied area. The ophiolitic mélange thrusted over gneiss rocks and metagabbro and both of them are intruded by mylonitized granite at W. Sikait.

**Fig. 3:**
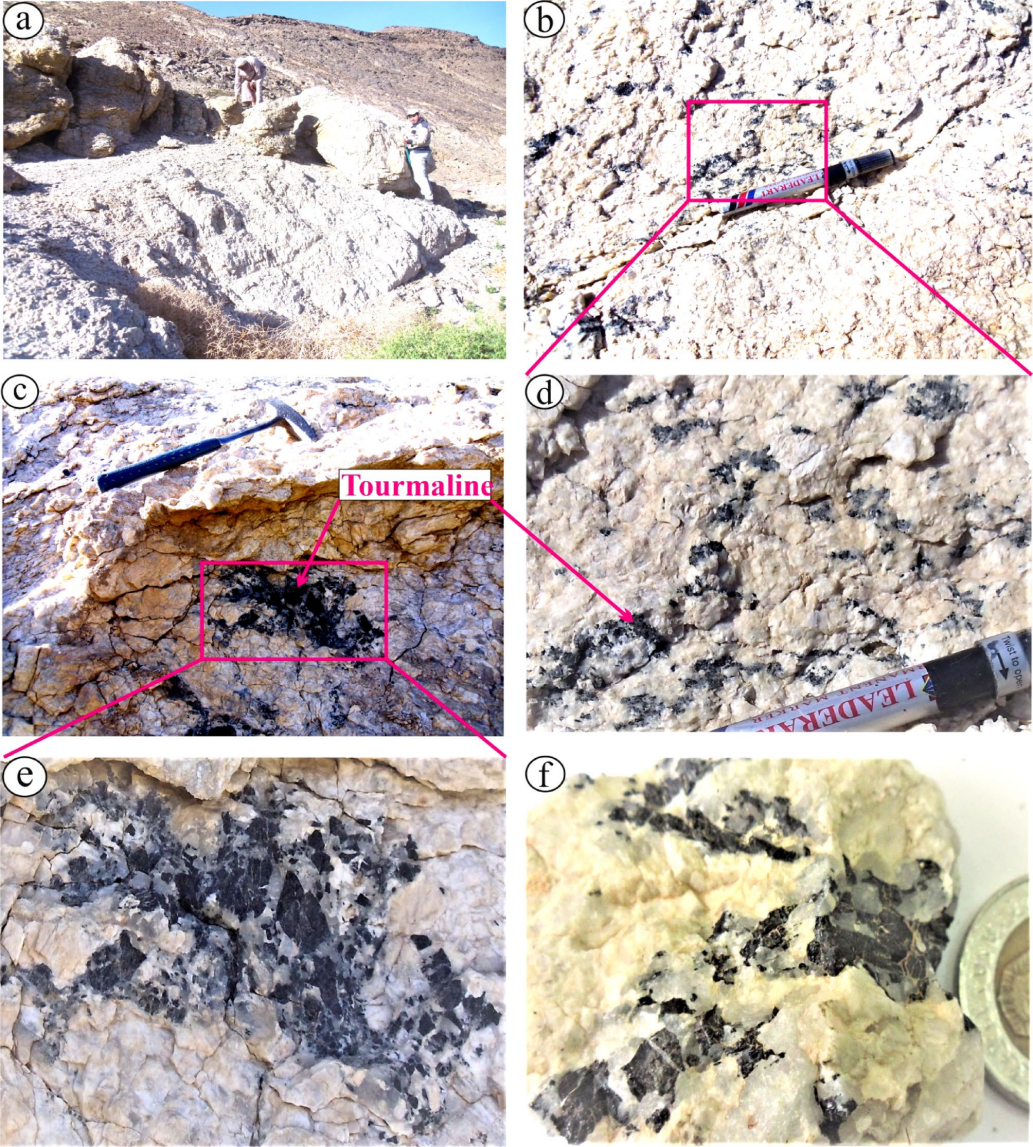
Field photographs reveal: (**a**) Large bouldaries on medium relief of tourmaline-bearing leucogranites; (**b**) Disseminated and separated tourmaline crystals within leucogranites; (**c**), (**d**), (**e**) & (**f**) disseminated and clustered nodules of well-developed tourmaline crystals disseminated within leucogranites.

Mylonitized granites (exposed along W. Abu Rusheid), alkali feldspar granites, and leucogranites are the main granitic rocks. Leucogranites granites occurs as white large (0.5 Km length and 250 m that are emplaced along NW-SE trend). They crop out as low to medium relief, offshoots of an irregular injected bodies intruding the ophiolitic mélange at W. Sikait (Fig. 3a). They are tourmaline-bearing granitic rocks. Tourmaline occurs as disseminated/isolated or cluster nodular within coarse-grained leucogranites (Figs. 3b, c, d, e and f). Light red alkali feldspar granites are exposed at the top right side of W. Sikait. They intrude the ophiolitic mélange and mylonitized granites at northern part of the studied area. Variable size xenoliths are observed in the alkali feldspar granites. Several post-granitic dikes such as pegmatite and quartz veins inject various granitic rocks.

## Methods and materials

Petrographical examination for thirteen samples of tourmaline-bearing leucogranites were performed out through plarizaing microscope at Nuclear Materials Authority. ICP-MS as well as XRF analyses for twenty two samples were performed at the Geo Analytical Lab, USA. Major and some trace elements were analyzed by Thermo ARL XRF Spectrometer (Precision and accuracy were controlled by analysis of International reference materials and replicate analyses and are 1% for major elements and 2 to 5% for trace and rare-earth elements(. In addition, REEs and some trace elements were measured using ICP-MS) 7700(. In Teflon containers that have been acid-washed, approximately 50 mg of powder from each sample is dissolved by refluxing in hot (250 °C) 3:1 nitric and hydrofluoric acid for a minimum of 8 h. The USGS standard rock powder GSP2, reference material 650 C, served as the calibration standard. The weight difference following ignition at 1000 °C was used to calculate loss on ignition (LOI). Mineral chemistry were analyzed using electron microprobe (a JEOL JXA-8500 F) at 15 kV voltage and 20 nA beam, and natural and synthetic mineral standards were used for calibration. The detection limits and analytical precision are listed on the laboratory’s website (https://environment.wsu.edu/facilities/geoanalytical-lab).

## Results

### Petrography

**Fig. 4:**
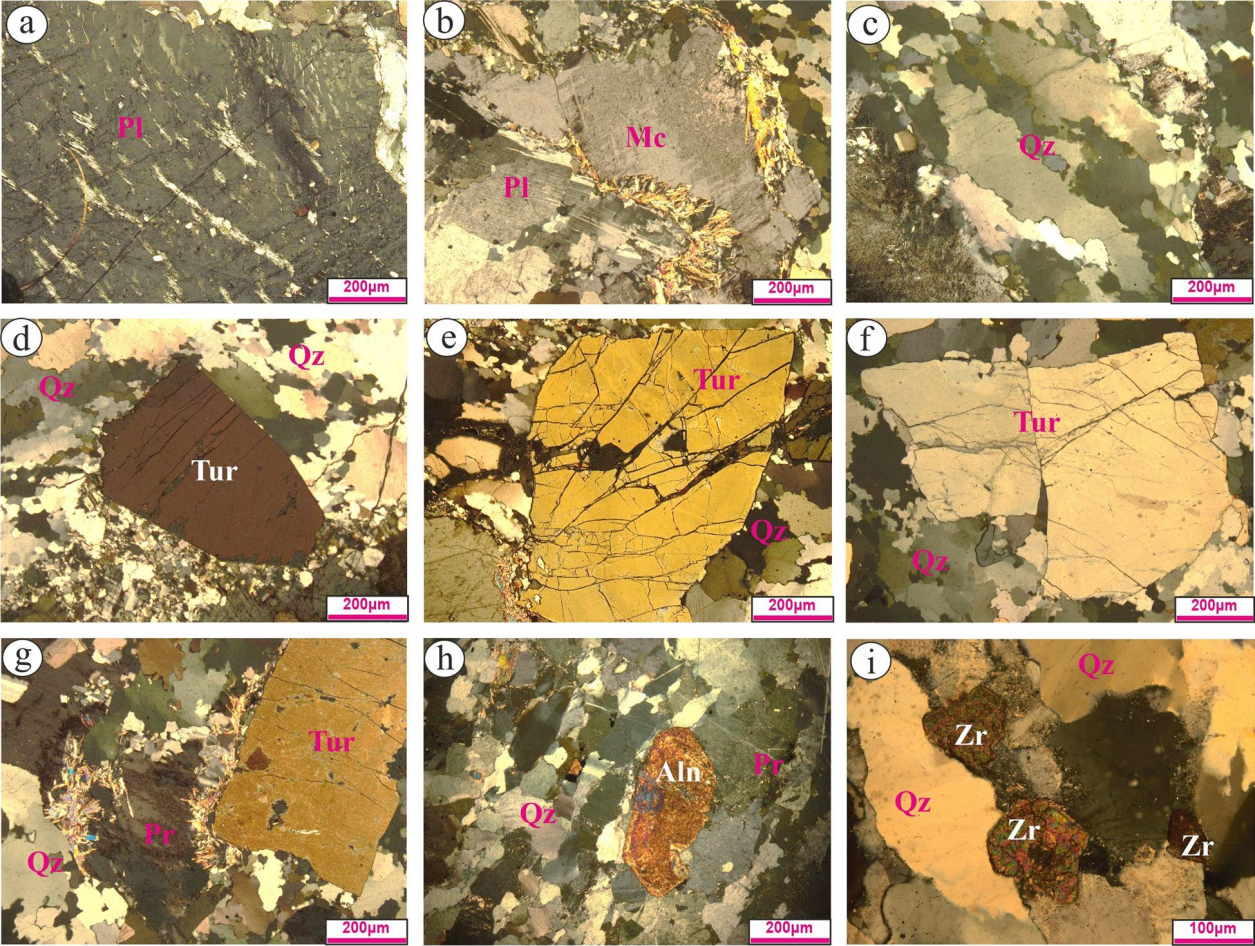
Photomicrographs (by Olympus X53 microscope) of tourmaline-bearing leucogranites exhibit: (**a**) Worm-like shape of string perthite (Pr); (**b**) Turbid microcline (Mc) mantled by sericite and associated twisted plagioclase (Pl); (**c**) Undulose extinsion of elongated quartz (Qz); (**d**) Well-developed tourmaline (Tur) crystal mantled by fine-grained quartz; (**e**), (** f**),(** g**) Coarse-grained of subhedral and fractured tourmaline crystals; h) Medium-grained of well-developed allanite (Aln) crystal; and (**i**) Two heart-like shape of zircon (Zr) crystals associated with quartz and perthite.

The examined tourmaline-bearing granitic rocks constitute mainly from plagioclase, K-feldspar, quartz, and with little muscovite amount. Tourmaline, zircon, and allanite consider the main accessories. The secondary include kaolinite, sericite, and chlorite. Potash Feldspar is represented by pristine to turbid crystals. It’s medium- grained microcline and flamy perthite with maximum length and width are 6.3 mm and 4.3 mm, respectively. Occasionally, they exhibit slightly to completely turbid surface due to kaolinitization and sericitization processes (Figs. 4a &b). Plagioclase is of albitic type that occurs as subhedral, and medium- grained crystals. Sometime, it’s deformed to the point of twisting and breaking. It exhibits lamellar twinning that is partially to completely disappear due to kaolinitization process. Fine sized grains of quartz as elongated and reveals wavy extinsion (Fig. 4c). Fine flaky of muscovite present commonly altered to sericite. Tourmaline occurs as well-developed euhedral to subhedral crystals, medium sized grains, and brownish- grey color with high relief crystals (Figs. 4d-g). Allanite occurs as brownish, medium sized grains, reaches up to 2.8 mm in length and 1.1 mm in width (Fig. 4h). Zircon occurs as fine-grained with heart like-shape. Occasionally, it reveals pleochroic halos due to its radioactive contents (Fig. 4i).

### Geochemistry

Geochemistry of twenty two samples from tourmaline-bearing leucogranites are listed in Table 1. The examined tourmaline-bearing leucogranites reveal wide variations in some major elements such as SiO_2_ (69.44–75.87 wt%, av. 72.38 wt%), and Fe_2_O_3_ (0.81–3.92 wt%, av. 1.92 wt%). They have high content of SiO_2_ (av. 72.38 wt%), total alkalis (av. 7.47 wt%), moderately Al_2_O_3_ (av. 15.09 wt%), and less MgO, CaO, MnO, TiO_2_ and Fe_2_O_3_ values (av. 0.15, 0.4, 0.2, 0.02, and 1.92 wt%, respectively).

**Table 1:** Whole rock (major,trace and rare Earth elements abundance) analysis of tourmaline- bearing leucogranites, Sikait, South Eastern Desert, egypt.

Sample No.	Gt1	Gt2	Gt3	Gt4	Gt5	Gt6	Gt7	Gt8	Gt9	Gt10	Gt11	Gt12	Gt13	Gt14	Gt15	Gt16	Gt17	Gt18	Gt19	Gt20	Gt21	Gt22
**SiO2**	74.18	75.41	69.44	71.58	75.87	74.54	70.51	69.85	72.94	73.08	72.26	71.14	70.61	72.56	74.06	73.02	71.81	72.01	74.03	72.14	69.66	71.78
**TiO2**	0.04	0.01	0.04	0.02	0.01	0.01	0.01	0.02	0.02	0.02	0.02	0.03	0.02	0.02	0.02	0.04	0.02	0.03	0.01	0.03	0.02	0.02
**Al2O3**	14.53	14.07	16.42	15.45	14.06	14.43	15.46	16.05	14.62	14.54	15.06	15.01	15.05	16.11	14.88	15.25	14.88	15.24	14.43	15.11	16.35	15.11
**Fe2O3**	1.41	1.12	2.43	1.71	0.81	0.93	1.03	1.39	1.98	2.03	2.35	3.75	3.92	1.31	1.15	2.07	2.64	2.08	1.51	3.03	1.47	2.24
**MnO**	0.05	0.04	0.04	0.03	0.04	0.06	0.04	0.05	0.04	0.05	0.05	1.01	1.06	0.04	0.04	0.06	0.52	0.17	0.09	0.69	0.06	0.22
**MgO**	0.11	0.11	0.24	0.14	0.09	0.11	0.07	0.16	0.19	0.17	0.19	0.18	0.20	0.12	0.12	0.21	0.21	0.15	0.11	0.19	0.18	0.17
**CaO**	0.51	0.51	0.42	0.32	0.57	0.58	0.25	0.27	0.28	0.29	0.29	0.40	0.36	0.49	0.52	0.48	0.45	0.28	0.32	0.46	0.51	0.29
**Na2O**	4.81	5.00	4.01	3.51	4.62	4.54	3.05	3.13	2.63	2.61	2.72	3.05	3.15	5.16	4.75	4.03	2.94	3.51	3.43	3.25	4.53	3.17
**K2O**	2.16	2.15	3.83	5.01	2.48	3.01	6.68	6.31	4.09	4.49	4.02	3.04	3.38	2.72	2.11	2.33	3.92	4.23	4.51	3.62	4.22	4.52
**P2O5**	0.07	0.06	0.15	0.11	0.14	0.08	0.12	0.15	0.13	0.15	0.14	0.06	0.09	0.09	0.06	0.12	0.17	0.11	0.09	0.17	0.20	0.16
**LOI**	0.55	0.61	0.57	0.61	0.49	0.61	0.52	0.49	0.56	0.62	0.67	0.47	0.41	0.62	0.74	0.75	0.58	0.61	0.54	0.62	0.66	0.57
**Total**	98.42	99.09	97.59	98.49	99.18	98.90	97.74	97.87	97.48	98.05	97.77	98.14	98.25	99.24	98.45	98.36	98.14	98.42	99.07	99.31	97.86	98.25
**Normative compositions**																						
**Qz**	37.78	37.888	31.031	31.788	39.38	36.398	27.107	27.263	41.714	40.469	40.778	41.274	38.866	31.965	38.185	40.357	39.122	35.189	36.662	38.837	26.899	35.797
**C**	4.28	3.47	5.61	4.25	3.78	3.65	3.21	4.07	5.87	5.39	6.23	6.51	6.04	4.68	4.78	6.10	5.56	4.89	3.91	5.62	4.33	5.00
**Or**	12.77	12.71	22.63	29.61	14.66	17.79	39.48	37.29	24.17	26.54	23.76	17.97	19.98	16.07	12.47	13.77	23.17	25.00	26.65	21.39	24.94	26.71
**Ab**	40.70	42.31	33.93	29.70	39.09	38.42	25.81	26.49	22.25	22.09	23.02	25.81	26.65	43.66	40.19	34.10	24.88	29.70	29.02	27.50	38.33	26.82
**An**	0.00	0.13	0.18	0.00	0.00	0.14	0.00	0.00	0.00	0.00	0.00	0.53	0.45	0.00	0.00	0.00	0.65	0.00	0.00	0.62	0.00	0.00
**Hy**	0.27	0.27	0.60	0.35	0.22	0.27	0.17	0.40	0.47	0.42	0.47	0.45	0.50	0.30	0.30	0.52	0.52	0.37	0.27	0.47	0.45	0.42
**Mt**	0.05	0.10	0.02	0.04	0.10	0.17	0.10	0.11	0.07	0.11	0.11	3.21	3.40	0.07	0.07	0.08	1.64	0.47	0.27	2.17	0.14	0.66
**Il**	0.08	0.02	0.08	0.04	0.02	0.02	0.02	0.04	0.04	0.04	0.04	0.06	0.04	0.04	0.04	0.08	0.04	0.06	0.02	0.06	0.04	0.04
**Hm**	1.38	1.05	2.42	1.68	0.74	0.82	0.96	1.32	1.93	1.96	2.28	1.54	1.57	1.26	1.10	2.02	1.51	1.76	1.33	1.54	1.38	1.79
**Ap**	0.17	0.14	0.36	0.26	0.33	0.19	0.28	0.36	0.31	0.36	0.33	0.14	0.21	0.21	0.14	0.28	0.40	0.26	0.21	0.40	0.47	0.38
**Fr**	0.58	0.56	0.26	0.24	0.54	0.62	0.13	0.10	0.15	0.13	0.15	0.30	0.21	0.52	0.61	0.45	0.13	0.19	0.28	0.15	0.34	0.11
**Sum**	98.05	98.65	97.11	97.96	98.86	98.48	97.27	97.43	96.98	97.48	97.16	97.78	97.93	98.78	97.90	97.75	97.62	97.88	98.62	98.76	97.32	97.73
**Trace elements (ppm)**																						
**F**	0.31	0.28	0.14	0.21	0.41	0.31	0.28	0.28	0.22	0.20	0.17	0.15	0.11	0.28	0.31	0.29	0.08	0.19	0.27	0.09	0.32	0.18
**Rb**	194.80	192.17	347.04	443.63	222.58	242.26	678.55	622.72	424.65	463.99	417.95	301.91	340.40	284.68	231.98	261.08	372.79	411.26	442.12	337.17	421.32	443.11
**Ba**	20.42	25.13	31.69	20.38	20.84	30.28	30.63	22.63	16.60	24.09	10.86	22.66	21.47	20.91	15.69	13.00	26.01	21.74	31.09	15.82	21.86	24.91
**Sr**	18.51	17.69	14.12	16.98	18.89	21.64	17.25	17.72	13.44	16.65	17.34	16.02	15.97	21.15	20.87	15.78	15.98	15.82	16.32	15.92	14.78	18.06
**Nb**	171.05	141.69	51.57	141.46	45.77	22.81	83.34	105.73	75.88	101.31	95.35	51.85	60.69	100.64	71.88	76.58	40.67	56.29	52.37	50.36	134.04	51.24
**Zr**	123.91	104.49	53.16	111.49	44.15	26.48	61.77	72.82	62.11	71.44	71.38	62.93	65.13	73.26	60.00	62.94	46.53	47.44	48.01	56.70	88.83	46.48
**Y**	5.86	6.25	5.06	< d.l.	8.52	12.30	3.46	5.46	3.49	4.26	4.34	64.07	75.13	8.66	10.65	6.71	43.04	8.68	5.86	44.76	5.62	12.43
**Zn**	221.86	148.47	419.44	291.22	116.74	121.60	181.68	269.48	356.63	340.66	422.63	304.33	283.28	202.75	184.78	341.73	255.33	315.65	228.52	283.06	270.11	307.07
**Cu**	< d.l.	1.34	1.24	< d.l.	1.00	< d.l.	1.17	< d.l.	1.33	< d.l.	1.24	1.00	< d.l.	1.24	1.11	1.00	1.02	< d.l.	1.24	1.19	2.04	2.01
**Ni**	< d.l.	< d.l.	1.07	< d.l.	< d.l.	1.11	3.53	3.88	< d.l.	< d.l.	1.00	< d.l.	< d.l.	1.17	1.01	< d.l.	1.11	< d.l.	1.06	2.04	1.35	1.47
**Co**	64.68	83.72	76.38	62.67	53.09	49.22	61.68	52.65	49.52	57.85	53.64	50.26	51.37	41.42	45.07	55.18	51.44	44.32	44.88	60.17	36.23	71.81
**Cr**	< d.l.	< d.l.	< d.l.	< d.l.	< d.l.	< d.l.	< d.l.	< d.l.	< d.l.	< d.l.	< d.l.	< d.l.	< d.l.	< d.l.	< d.l.	< d.l.	< d.l.	< d.l.	< d.l.	1.11	< d.l.	1.07
**V**	9.14	89.09	8.84	11.87	8.71	9.27	10.80	11.60	8.87	10.21	13.72	9.22	13.01	11.15	11.04	6.45	10.04	9.02	11.64	8.55	11.89	11.27
**Hf**	0.89	0.63	1.49	2.47	0.61	0.61	0.79	0.80	1.25	1.12	1.41	2.11	1.89	0.15	0.53	1.07	1.26	1.08	1.19	1.72	1.03	1.88
**Ta**	31.44	21.51	11.95	29.05	8.04	3.69	24.77	34.77	35.64	39.68	36.50	16.68	16.92	16.42	12.44	17.92	6.63	13.70	12.22	9.54	23.99	11.06
**Pb**	18.69	16.91	13.91	20.74	18.94	21.54	32.30	25.60	29.39	235.39	19.37	12.43	15.90	16.23	17.41	19.71	18.17	19.41	19.33	15.78	21.65	11.98
**Th**	5.12	3.65	5.59	7.46	2.14	5.17	11.90	8.71	6.25	7.95	9.63	16.62	15.05	8.02	6.75	6.07	7.25	6.65	9.80	8.63	7.89	6.66
**U**	1.22	1.23	1.28	1.42	1.21	1.27	1.64	1.39	1.69	1.52	1.54	3.52	3.52	1.33	1.68	1.39	2.37	1.03	1.07	2.36	1.14	1.32
**Sc**	0.37	< d.l.	0.54	< d.l.	< d.l.	0.97	< d.l.	< d.l.	< d.l.	< d.l.	< d.l.	3.10	5.31	0.16	1.80	< d.l.	2.35	0.47	0.46	3.24	< d.l.	< d.l.
**Ga**	22.00	20.37	29.72	23.53	16.83	18.76	24.21	28.89	28.30	28.54	30.23	27.06	26.92	< d.l.	24.23	26.64	24.86	23.99	23.39	32.70	< d.l.	23.26
**Sn**	13.19	14.87	11.58	10.62	11.57	14.51	18.47	18.80	23.43	20.85	19.68	17.18	18.09	16.88	25.16	21.96	19.66	13.55	13.21	21.50	8.17	14.38
**Cs**	16.40	15.25	30.59	37.97	18.09	18.55	45.89	41.24	26.58	30.73	27.77	27.38	32.81	24.83	21.65	22.27	31.32	36.22	35.32	35.70	54.49	35.27
**Rare earth elements (ppm)**																						
**La**	1.29	1.12	1.24	0.64	1.72	1.54	0.93	1.30	1.58	1.57	1.62	4.05	5.63	1.85	2.13	1.61	2.80	1.28	1.25	4.38	1.07	1.62
**Ce**	3.04	2.65	3.00	1.47	4.16	3.71	2.08	3.14	3.48	3.60	3.75	11.02	14.65	4.03	5.29	4.00	7.39	3.16	3.08	11.61	1.87	4.22
**Pr**	0.42	0.36	0.39	0.21	0.59	0.52	0.27	0.44	0.47	0.46	0.50	1.61	2.06	0.62	0.72	0.54	1.12	0.45	0.44	1.66	0.33	0.57
**Nd**	1.79	1.35	1.74	0.82	2.35	2.30	0.98	1.64	1.75	1.87	1.75	6.52	8.48	2.74	2.89	2.08	4.36	1.92	1.87	6.82	1.42	2.36
**Sm**	1.12	0.88	1.07	0.55	1.39	1.38	0.66	1.08	0.99	0.89	0.95	4.43	5.27	1.11	1.49	1.13	2.85	1.11	1.08	4.18	0.61	1.31
**Eu**	0.01	0.01	0.02	0.00	0.02	0.03	0.01	0.01	0.01	0.01	0.00	0.00	0.01	0.00	0.02	0.02	0.01	0.01	0.01	0.01	0.00	0.01
**Gd**	0.99	0.86	0.95	0.45	1.38	1.41	0.60	0.92	0.94	0.82	0.81	5.97	6.78	1.29	1.31	1.18	4.11	1.33	0.94	4.67	0.90	1.90
**Tb**	0.16	0.15	0.17	0.09	0.28	0.29	0.10	0.20	0.15	0.16	0.15	1.67	1.90	0.20	0.21	0.19	1.17	0.33	0.21	1.30	0.14	0.45
**Dy**	0.80	0.90	0.94	0.46	1.61	1.77	0.52	1.07	0.84	0.86	0.86	10.80	11.51	1.27	1.32	1.11	7.07	1.73	0.99	7.26	1.01	2.35
**Ho**	0.13	0.14	0.12	0.07	0.23	0.31	0.09	0.14	0.13	0.12	0.11	1.40	1.53	0.19	0.20	0.18	0.87	0.20	0.12	0.88	0.11	0.32
**Er**	0.37	0.41	0.31	0.15	0.69	1.01	0.25	0.37	0.35	0.37	0.36	3.15	3.47	0.58	0.58	0.48	1.96	0.46	0.28	2.01	0.32	0.67
**Tm**	0.07	0.08	0.05	0.03	0.11	0.22	0.05	0.06	0.07	0.06	0.07	0.50	0.57	0.09	0.11	0.09	0.30	0.07	0.04	0.32	0.04	0.11
**Yb**	0.64	0.65	0.40	0.20	0.96	1.59	0.41	0.47	0.46	0.58	0.54	3.76	4.28	0.88	0.93	0.69	2.22	0.55	0.35	2.17	0.44	0.80
**Lu**	0.10	0.09	0.05	0.03	0.15	0.24	0.06	0.07	0.07	0.08	0.08	0.44	0.50	0.10	0.14	0.10	0.24	0.07	0.07	0.24	0.06	0.10
**Geochemical parameters**																						
**LREEs**	7.65	6.36	7.44	3.68	10.22	9.44	4.92	7.60	8.27	8.39	8.56	27.63	36.09	10.34	12.52	9.36	18.51	7.92	7.72	28.65	5.29	10.07
**HREEs**	3.27	3.28	3.01	1.49	5.44	6.85	2.07	3.29	3.02	3.06	2.98	27.68	30.54	4.60	4.82	4.03	17.94	4.73	3.00	18.86	3.01	6.70
**LREEs/HREEs**	2.34	1.94	2.47	2.48	1.88	1.38	2.38	2.31	2.74	2.75	2.88	1.00	1.18	2.25	2.60	2.33	1.03	1.67	2.58	1.52	1.76	1.50
**∑REEs**	10.92	9.64	10.45	5.17	15.66	16.29	7.00	10.89	11.29	11.45	11.54	55.31	66.64	14.94	17.34	13.39	36.46	12.65	10.72	47.51	8.31	16.77
Eu/Eu*	0.03	0.04	0.06	0.02	0.04	0.06	0.04	0.03	0.02	0.04	0.00	0.00	0.00	0.00	0.04	0.04	0.01	0.02	0.01	0.01	0.01	0.01
**(La/Yb)cn**	1.37	1.17	2.11	2.17	1.21	0.65	1.53	1.88	2.33	1.84	2.04	0.73	0.89	1.42	1.55	1.58	0.85	1.57	2.43	1.36	1.65	1.36
**(Tb/Yb)cn**	1.09	0.99	1.88	1.99	1.25	0.78	0.99	1.81	1.42	1.17	1.22	1.90	1.89	0.95	0.97	1.17	2.26	2.53	2.53	2.57	1.40	2.39
**(Gd/Yb)cn**	1.26	1.07	1.93	1.85	1.16	0.72	1.19	1.59	1.65	1.14	1.21	1.28	1.28	1.18	1.14	1.39	1.50	1.93	2.18	1.74	1.67	1.91
**(La/Sm)cn**	0.72	0.81	0.73	0.74	0.78	0.70	0.89	0.76	1.01	1.12	1.07	0.58	0.67	1.04	0.90	0.90	0.62	0.73	0.73	0.66	1.11	0.78
Ce/Ce*	1.01	1.06	1.03	1.01	1.04	1.01	1.05	1.07	1.02	1.03	1.08	1.11	1.09	0.91	1.07	1.09	1.09	1.03	1.03	1.09	0.76	1.10
**Pr/Pr’**	1.03	1.13	0.98	1.12	1.10	1.02	1.12	1.15	1.10	1.04	1.16	1.14	1.10	1.02	1.08	1.12	1.17	1.06	1.06	1.11	1.00	1.07
**Tb/Tb’**	1.16	1.15	1.32	1.34	1.32	1.21	1.07	1.45	1.13	1.30	1.30	1.62	1.64	1.03	1.09	1.07	1.70	1.66	1.54	1.74	1.15	**1.53**
**Dy/Dy’**	1.09	1.22	1.39	1.19	1.31	1.17	1.08	1.42	1.16	1.28	1.35	1.63	1.57	1.22	1.23	1.12	1.65	1.58	1.39	1.62	1.57	1.40
**T1**	1.02	1.10	1.01	1.07	1.07	1.02	1.08	1.11	1.06	1.03	1.12	1.13	1.09	0.97	1.08	1.11	1.13	1.04	1.04	1.10	0.87	1.09
**T3**	1.13	1.19	1.35	1.26	1.31	1.19	1.07	1.43	1.15	1.29	1.33	1.63	1.60	1.12	1.16	1.10	1.68	1.62	1.46	1.68	1.34	1.46
**TE1**,**3**	1.07	1.14	1.17	1.16	1.19	1.10	1.08	1.26	1.10	1.16	1.22	1.35	1.32	1.04	1.12	1.10	1.38	1.30	1.23	1.36	1.08	1.26
**tau1**	1.06	1.25	0.90	1.15	1.12	0.78	1.32	1.29	1.17	0.91	1.30	1.27	1.15	0.69	0.91	1.12	1.43	1.20	1.13	1.15	1.01	1.28
**tau2**	0.48	0.55	0.45	0.61	0.46	0.44	0.59	0.60	0.48	0.34	0.52	0.41	0.36	0.15	0.45	0.41	0.35	0.28	0.42	0.40	0.05	0.20
**tau3**	0.12	0.18	0.32	0.35	0.28	0.22	0.10	0.39	0.12	0.31	0.30	0.61	0.61	0.15	0.19	0.12	0.62	0.58	0.47	0.63	0.28	0.47
**tau4**	0.41	0.48	0.44	0.39	0.31	0.47	0.45	0.37	0.41	0.43	0.56	0.39	0.43	0.40	0.37	0.36	0.47	0.43	0.14	0.49	0.24	0.40
**Ce_MFR**	1.15	1.11	1.20	1.07	1.09	1.10	1.13	1.09	1.08	1.15	1.09	1.12	1.14	1.00	1.11	1.13	1.09	1.15	1.14	1.13	0.88	1.20
**Eu_MFR**	0.02	0.03	0.05	-	0.03	0.05	0.04	0.02	0.02	0.03	-	-	0.00	-	0.04	0.04	0.01	0.02	0.02	0.01	-	0.02
**Mg/(Mg + Fe)**	13.39	16.29	16.36	13.96	18.04	18.98	11.87	18.57	15.97	14.23	13.81	8.68	9.18	15.36	17.13	16.73	13.61	12.50	12.61	11.05	19.52	13.07
**K2O/Na2O**	0.45	0.43	0.96	1.43	0.54	0.66	2.19	2.02	1.56	1.72	1.48	1.00	1.07	0.53	0.44	0.58	1.33	1.21	1.31	1.11	0.93	1.43
**Na2O/K2O**	2.23	2.33	1.05	0.70	1.86	1.51	0.46	0.50	0.64	0.58	0.68	1.00	0.93	1.90	2.25	1.73	0.75	0.83	0.76	0.90	1.07	0.70
**R1**	2690.75	2715.56	2243.54	2306.44	2811.76	2624.81	2025.72	2030.53	2917.46	2839.20	2847.32	2849.18	2694.98	2330.87	2722.75	2834.15	2755.36	2507.72	2619.99	2727.34	2007.07	2541.60
**R2**	345.04	336.02	378.93	344.24	341.25	350.57	333.48	351.66	326.17	324.67	335.87	346.16	343.66	374.39	353.47	360.91	350.45	336.34	322.75	355.04	384.21	335.85
**Y/Nb**	0.03	0.04	0.10	-	0.19	0.54	0.04	0.05	0.05	0.04	0.05	1.24	1.24	0.09	0.15	0.09	1.06	0.15	0.11	0.89	0.04	0.24
**Rb/Nb**	1.14	1.36	6.73	3.14	4.86	10.62	8.14	5.89	5.60	4.58	4.38	5.82	5.61	2.83	3.23	3.41	9.17	7.31	8.44	6.70	3.14	8.65
**Rb/Sr**	10.52	10.86	24.58	26.13	11.78	11.20	39.34	35.14	31.60	27.87	24.10	18.85	21.31	13.46	11.12	16.54	23.33	26.00	27.09	21.18	28.51	24.54
**Nb/Ta**	5.44	6.59	4.32	4.87	5.69	6.18	3.36	3.04	2.13	2.55	2.61	3.11	3.59	6.13	5.78	4.27	6.13	4.11	4.29	5.28	5.59	4.63
**Y/Ho**	46.88	45.96	44.00	-	36.72	39.30	40.23	40.15	27.48	35.21	38.41	45.93	49.07	46.06	54.62	37.49	49.30	43.84	47.64	50.98	52.04	39.34
**Zr/Hf**	139.22	165.86	35.68	45.14	72.38	43.41	78.19	91.03	49.69	63.79	50.62	29.82	34.46	488.40	113.21	58.82	36.93	43.93	40.34	32.97	86.24	24.72
**La/Nb**	0.01	0.01	0.02	0.00	0.04	0.07	0.01	0.01	0.02	0.02	0.02	0.08	0.09	0.02	0.03	0.02	0.07	0.02	0.02	0.09	0.01	0.03
**La/Ta**	0.04	0.05	0.10	0.02	0.21	0.42	0.04	0.04	0.04	0.04	0.04	0.24	0.33	0.11	0.17	0.09	0.42	0.09	0.10	0.46	0.04	0.15
**Na**	155.21	161.35	129.40	113.26	149.08	146.50	98.42	101.00	84.87	84.22	87.77	98.42	101.65	166.51	153.28	130.04	94.87	113.26	110.68	104.87	146.18	102.29
**K**	45.86	45.65	81.32	106.38	52.66	63.91	141.83	133.98	86.84	95.33	85.36	64.55	71.77	57.75	44.80	49.47	83.23	89.81	95.76	76.86	89.60	95.97
**Al**	285.01	275.99	322.08	303.06	275.79	283.05	303.25	314.83	286.78	285.21	295.41	294.43	295.21	316.00	291.88	299.13	291.88	298.94	283.05	296.39	320.71	296.39
**Rb/Nb**	1.14	1.36	6.73	3.14	4.86	10.62	8.14	5.89	5.60	4.58	4.38	5.82	5.61	2.83	3.23	3.41	9.17	7.31	8.44	6.70	3.14	8.65
**Rb/Ba**	9.54	7.65	10.95	21.77	10.68	8.00	22.15	27.52	25.58	19.26	38.49	13.32	15.85	13.61	14.79	20.08	14.33	18.92	14.22	21.31	19.27	17.79
**Sr/Ba**	0.91	0.70	0.45	0.83	0.91	0.71	0.56	0.78	0.81	0.69	1.60	0.71	0.74	1.01	1.33	1.21	0.61	0.73	0.52	1.01	0.68	0.73
**Rb/Zr**	1.57	1.84	6.53	3.98	5.04	9.15	10.99	8.55	6.84	6.49	5.86	4.80	5.23	3.89	3.87	4.15	8.01	8.67	9.21	5.95	4.74	9.53
**Sr/Y**	3.16	2.83	2.79	-	2.22	1.76	4.99	3.25	3.85	3.91	4.00	0.25	0.21	2.44	1.96	2.35	0.37	1.82	2.78	0.36	2.63	1.45
**Zr/Hf**	139.22	165.86	35.68	45.14	72.38	43.41	78.19	91.03	49.69	63.79	50.62	29.82	34.46	488.40	113.21	58.82	36.93	43.93	40.34	32.97	86.24	24.72
**U/Th**	0.24	0.34	0.23	0.19	0.57	0.25	0.14	0.16	0.27	0.19	0.16	0.21	0.23	0.17	0.25	0.23	0.33	0.15	0.11	0.27	0.14	0.20
**Agpaitic index (AI)**	0.71	0.75	0.65	0.72	0.73	0.74	0.79	0.75	0.60	0.63	0.59	0.55	0.59	0.71	0.68	0.60	0.61	0.68	0.73	0.61	0.74	0.67
**Al2O3/TiO2**	363.25	1407.00	410.50	772.50	1406.00	1443.00	1546.00	802.50	731.00	727.00	753.00	500.33	752.50	805.50	744.00	381.25	744.00	508.00	1443.00	503.67	817.50	755.50
**FeO + MgO**	1.52	1.23	2.67	1.85	0.90	1.04	1.10	1.55	2.17	2.20	2.54	3.93	4.12	1.43	1.27	2.28	2.85	2.23	1.62	3.22	1.65	2.41
**Al2O3/FeO + MgO**	9.56	11.44	6.15	8.35	15.62	13.88	14.05	10.35	6.74	6.61	5.93	3.82	3.65	11.27	11.72	6.69	5.22	6.83	8.91	4.69	9.91	6.27
**FeO/MgO**	12.82	10.18	10.13	12.21	9.00	8.45	14.71	8.69	10.42	11.94	12.37	20.83	19.60	10.92	9.58	9.86	12.57	13.87	13.73	15.95	8.17	13.18
**CaO/FeO + MgO**	0.04	0.05	0.04	0.03	0.06	0.07	0.02	0.03	0.03	0.02	0.02	0.02	0.02	0.04	0.05	0.05	0.04	0.02	0.02	0.03	0.06	0.02
**Na2O/K2O**	2.23	2.33	1.05	0.70	1.86	1.51	0.46	0.50	0.64	0.58	0.68	1.00	0.93	1.90	2.25	1.73	0.75	0.83	0.76	0.90	1.07	0.70
**K2O/Na2O**	0.45	0.43	0.96	1.43	0.54	0.66	2.19	2.02	1.56	1.72	1.48	1.00	1.07	0.53	0.44	0.58	1.33	1.21	1.31	1.11	0.93	1.43
**CaO/Na2O**	0.11	0.10	0.10	0.09	0.12	0.13	0.08	0.09	0.11	0.11	0.11	0.13	0.11	0.09	0.11	0.12	0.15	0.08	0.09	0.14	0.11	0.09
**Na2O + K2O**	6.97	7.15	7.84	8.52	7.10	7.55	9.73	9.44	6.72	7.10	6.74	6.09	6.53	7.88	6.86	6.36	6.86	7.74	7.94	6.87	8.75	7.69
**TZr(°C)**	786.30	767.70	720.00	775.30	701.40	663.20	720.80	737.10	742.80	750.80	754.60	745.30	744.50	740.10	729.20	740.70	716.00	712.50	709.60	730.40	750.80	711.90

**Fig. 5:**
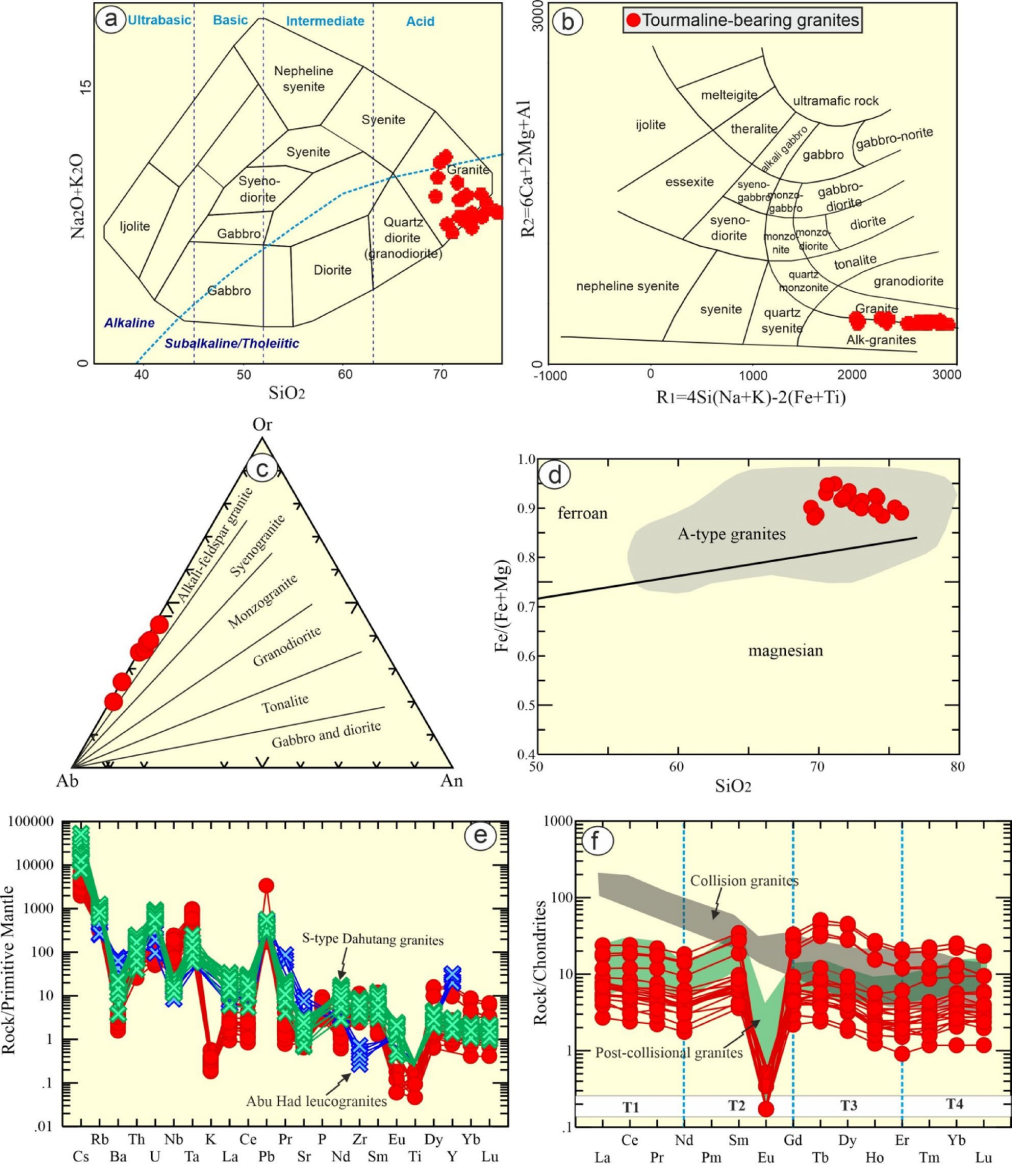
Bulk rock geochemical diagrams: (**a**) TAS diagram^32^; (**b**) Multi-cationic^33^; (**c**) Ab-Qz-An ternary normative^34^; (**d**)SiO_2_-Fe(Fe/Mg)^35^; (**e**) Trace patterns (primitive mantle)^36^. Abu Had S-type granites^38^ and highly fractionated S-type Dahutang granites, Southeast China^39^ respectively are used for comparison; and (**f**) REEs pattern (Chondrite)^36^. Dark gray and green fields are from^40^ and^41^ for comparison. T1, T2, T3, and T4 are four groups of tetrad effect^42^. Figs. from 3 to 10 have been drawn by Coreldrow program version 2021).

The examined rocks are discriminated by using several classification diagrams. According to TAS diagram^32^ the examined rocks lie in the field of granite (Fig. 5a), and alkali granites according to multicationic^33^ diagram (Fig. 5b). For further constraints, they plot in alkali feldspar granite by using their normative composition^34^ (Fig. 5c). The examined rocks is dominated by plagioclase (anorthite + albite), quartz, and orthoclase with less corundum and hypersthene, apatite, fluorite and iron oxides (magnetite + hematite) as a normative composition. The examined samples have less ratios of K_2_O/Na_2_O (0.43–2.19, avg. 1.1) and low Mg# [Mg/(Mg + Fe^T^)∗100] that ranges from 8.68 to 19.52 with an average 14.58. Their Fe# ranges from 0.79 to 0.95, suggesting a ferroan signature^35^ (Fig. 5d).

**Fig. 6:**
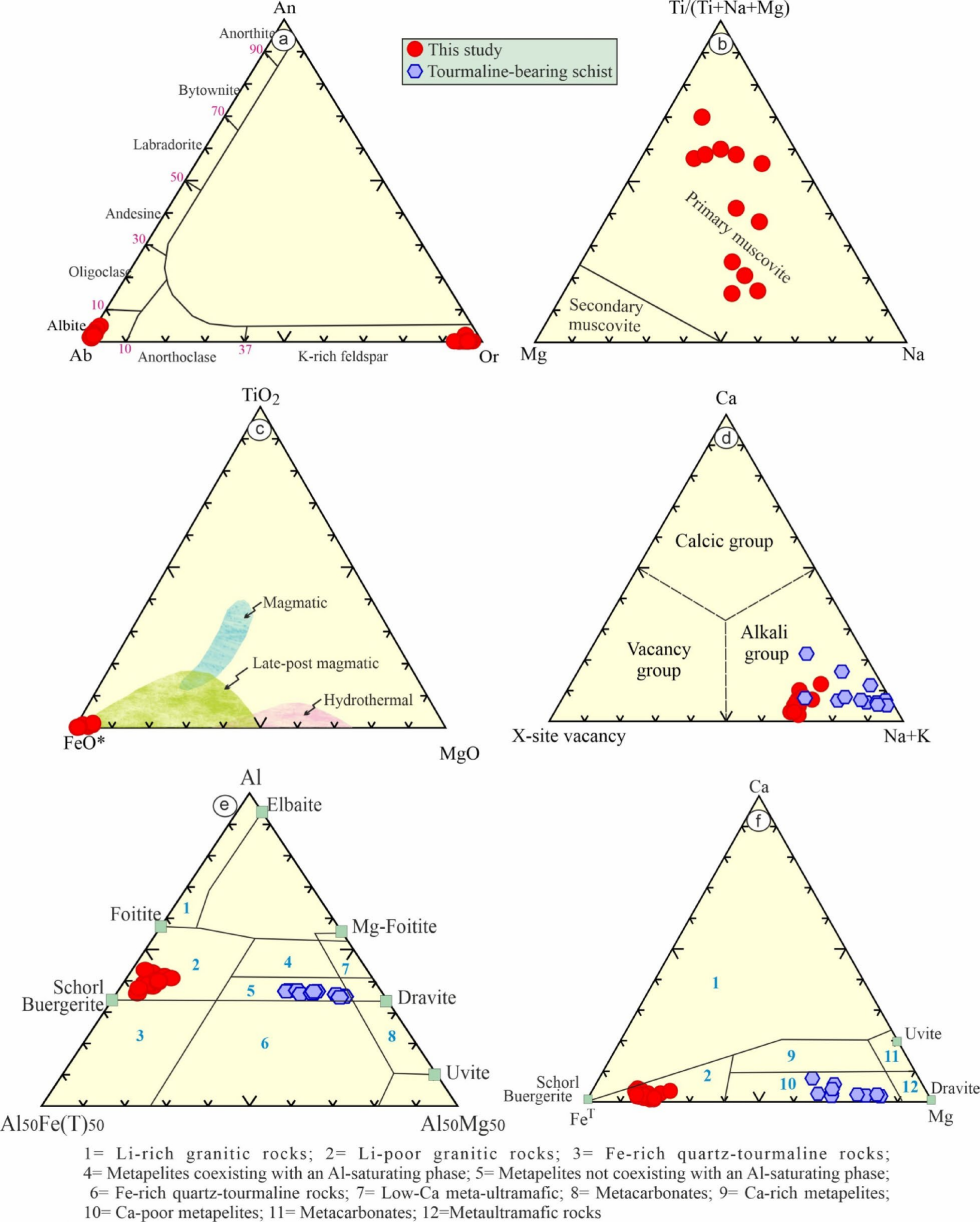
Mineral chemistry diagrams: (**a**) Ab-An-Or feldspar diagram^54^; (**b**) Muscovite type^50^; (**c**) FeO*-TiO_2_–MgO (wt%) for muscovite^51^**d**) Tourmaline dividing based on X-site^52^; (**e**) Fe^T^-Al-Mg diagram for tourmaline^53^ and **f**) Fe(Tot)-Ca-Mg ternary diagram for tourmaline.

The investigated samples possess high concentrations of elements such as Pb (11.98–235.39 ppm) (Table 1). Furthermore, they contain higher contents of LFSEs such as Rb (192.17- 678.55 ppm) and low Ba (av. 22.21 ppm) and Sr (av. 17.13 ppm). In addition, low transition element concentrations (av. 1.65, and 1.09 ppm for Ni, Cr, respectively) were recorded. Significant concentrations HFSEs such as Nb (av. 81 ppm), Zn (116.74–422.63 ppm) and Zr (av. 66.43 ppm) were recorded. The patterns^36^ of the examined rocks reveal notable strong positive of semi-volatile elements (Pb), enrichment Rb, K relative to Zr, U and Nb, as well as strong negative barium, titanium, and strontium anomalies (Fig. 6a), pointing fractionation of apatite, feldspars, and Fe-Ti oxides. These are characteristics of post-collisional granites^37^.

The examined rock patterns are very similar to Egyptian Abu Had S-type granites^38^ and highly fractionated S-type Dahutang granites, Southeast China^39^ as shown in Fig. 5e. However, they are similar to those of A-type granites of Baijuhuajian (South China)^43^ and Yangmeiwan granite^44^, but the examined rocks are depleted in ∑REEs. REEs concentration of the investigated samples are listed in Table 1. ∑REEs (av. 19.1 ppm) is low relative to the worldwide value^45^. The examined rocks have slightly notable depletion of HREEs (av. 7.44 ppm) in comparison with LREEs (av. 11.67 ppm). This is may be related to relative presence of some accessories certainly zircon mineral^46^. REE multi-elements^36^, (Fig. 5f). They exhibit parallel, uniform patterns of REEs, closely similar LREEs and HREEs pattern, low LREEs fractionated patterns (av. La/Sm)_Cn_ = 0.82) relative to those of HREEs (av. Gd/Yb)_Cn_=1.45) and moderate fractionation of LREE/HREE (La/Yb)_Cn_ = 0.65–2.4). Furthermore, they reveal extreme pronounced Eu* (av. 0.02, where Eu/Eu*= (Eu_N_/[Sm_N_ x Gd_N_]^0 [5].^) negative anomaly, which is consistent with Sr and Ba anomalies that are ascribed to extensive fractional crystallization as well as post-magmatic metasomatism^47–49^. In addition, the examined samples have positive Ce/Ce* = (Ce_N_/((La_N_)^2/3^ x (Nd_N_)^1/3^) anomaly (0.76–1.12; av. 1.04).

## Mineral chemistry

Variable minerals (from tourmaline-bearing leucogranites, Sikait, south Eastern Desert, Egypt) such as K-feldspar, albite, muscovite, zircon, tourmaline, apatite, goethite and magnetite were analyzed using Electron Microprobe Analysis.

### Feldspars

Tables 2 and 3 provide the EMPA of K-feldspars and albite from the examined tourmaline-bearing leucogranites (Fig. 6a). The microprobe of twenty three spot analysis of K-feldspars reveal a narrow variations in their composition with SiO_2_ ranges from 64.31 wt% to 65.52 wt%, K_2_O from 15.76 wt% to 16.38 wt% and Al_2_O_3_ from 18.02 wt% to 18.43 wt% and very low Na_2_O (av. 0.45 wt%). Their structural has been calculated computed and yielded composition with end member Or_(93−98)_Ab _(2−7)_An _(0−2)_. On contrary, the analyzed plagioclase (24 spot analysis) are typically albite with end member Or_(0.4−1)_Ab _(94−98)_An _(1−5)_.

**Table 2:** Electron microprobe analyses of K-feldspar from tourmaline - bearing leucogranites, sikait, South Eastern desert, egypt.

Mineral	K-Feldspars																						
Spot No.	#1	#2	#3	#4	#5	#6	#7	#8	#9	#10	#11	#13	#14	#15	#16	#17	#18	#19	#20	#21	#22	#23	#23
**SiO2**	64.56	64.78	65.43	64.65	64.87	65.52	64.33	64.92	65.26	64.88	65.12	64.58	64.90	65.16	64.84	64.59	64.38	64.31	64.83	65.08	64.73	65.07	64.52
**TiO2**	0.00	0.00	0.00	0.00	0.00	0.04	0.00	0.01	0.02	0.00	0.00	0.02	0.01	0.00	0.00	0.00	0.00	0.00	0.00	0.01	n.d.	0.02	0.00
**Al2O3**	18.43	18.21	18.28	18.25	18.42	18.23	18.27	18.16	18.25	18.25	18.38	18.21	18.05	18.11	18.17	18.16	18.33	18.29	18.12	18.02	18.05	18.43	18.41
FeO*	0.00	0.00	0.02	0.00	0.03	0.03	0.02	0.00	0.03	0.00	0.00	0.00	0.00	0.00	0.00	0.04	0.00	0.03	0.12	0.03	0.02	0.02	0.00
**MnO**	0.00	0.00	0.00	0.00	0.00	0.00	0.00	0.00	0.01	0.00	0.00	0.00	0.00	0.00	0.00	0.00	0.00	0.00	0.00	0.00	0.00	0.00	0.00
**MgO**	0.00	0.00	0.00	0.01	0.02	0.00	0.00	0.00	0.00	0.00	0.00	0.00	0.00	0.01	0.00	0.00	0.00	0.00	0.03	0.00	0.00	0.01	0.00
**CaO**	0.01	0.01	0.02	0.01	0.01	0.04	0.01	0.03	0.02	0.02	0.05	0.02	0.01	0.02	0.41	0.03	0.03	0.02	0.01	0.01	0.02	0.02	0.01
**Na2O**	0.65	0.65	0.26	0.63	0.54	0.31	0.38	0.38	0.32	0.66	0.47	0.39	0.42	0.36	0.35	0.42	0.35	0.38	0.41	0.45	0.38	0.77	0.39
**K2O**	15.94	15.93	16.38	15.93	16.15	16.29	16.19	16.29	16.26	15.76	16.09	16.34	16.22	16.24	16.08	16.04	16.29	16.24	16.31	16.12	16.16	15.77	16.13
**P2O5**	0.05	0.04	0.00	0.00	0.00	0.00	0.15	0.13	0.07	0.13	0.15	0.15	0.12	0.00	0.34	0.07	0.00	0.00	0.00	0.00	0.00	0.02	0.02
**Cr2O3**	0.00	0.03	0.03	0.00	0.00	0.06	0.00	0.00	0.01	0.00	0.00	n.d.	0.02	0.01	0.02	0.01	0.00	0.00	0.00	0.02	0.00	0.00	0.00
**SO3**	0.00	0.00	0.01	0.00	0.00	0.00	0.00	0.00	0.00	0.00	0.00	0.00	0.00	0.00	0.00	0.00	0.00	0.00	0.00	0.00	0.00	0.00	0.00
**NiO**	0.00	0.00	0.01	0.00	0.00	0.00	0.00	0.00	0.00	0.00	0.00	0.00	0.00	0.00	0.00	0.03	0.00	0.00	0.00	0.02	0.00	0.01	0.00
**B2O3**	0.00	0.00	0.00	0.00	0.00	0.00	0.00	0.00	0.00	0.00	0.00	0.00	0.00	0.00	0.00	0.00	0.00	0.00	0.00	0.00	0.00	0.00	0.01
**ZrO2**	0.00	0.00	0.00	0.00	0.00	0.00	0.00	0.00	0.00	0.00	0.01	0.00	0.00	0.00	0.00	0.00	0.00	0.00	0.01	0.00	0.00	0.00	0.00
**Nb2O5**	0.00	0.00	0.02	0.00	0.00	0.00	0.00	0.00	0.00	0.00	0.00	0.00	0.00	0.00	0.00	0.00	0.00	0.00	0.00	0.00	0.00	0.00	0.00
**Ta2O5**	0.00	0.00	0.00	0.00	0.00	0.04	0.00	0.00	0.07	0.00	0.00	0.00	0.00	0.00	0.00	0.00	0.00	0.00	0.00	0.02	0.00	0.00	0.00
**SnO2**	0.00	0.00	0.00	0.00	0.00	0.00	0.00	0.00	0.00	0.00	0.00	0.00	0.00	0.00	0.01	0.00	0.00	0.00	0.00	0.00	0.00	0.00	0.00
**HfO2**	0.00	0.00	0.00	0.00	0.00	0.00	0.00	0.00	0.00	0.00	0.00	0.00	0.00	0.00	0.00	0.00	0.00	0.00	0.00	0.00	0.00	0.00	0.00
**ThO2**	0.00	0.00	0.00	0.00	0.00	0.00	0.00	0.00	0.00	0.00	0.00	0.00	0.00	0.00	0.00	0.00	0.00	0.00	0.00	0.00	0.00	0.00	0.00
**Total**	99.64	99.65	100.46	99.48	100.04	100.56	99.35	99.92	100.32	99.70	100.27	99.71	99.76	99.91	100.22	99.39	99.38	99.27	99.84	99.80	99.36	100.14	99.49
**Cations on the basis of 8 oxygen**																							
**Si**	2.99	3.00	3.01	3.00	3.00	3.01	3.00	3.01	3.01	3.00	3.00	3.00	3.01	3.01	3.00	3.00	3.00	3.00	3.00	3.01	3.01	3.00	3.00
**Al**	1.01	1.00	0.99	1.00	1.00	0.99	1.00	0.99	0.99	1.00	1.00	1.00	0.99	0.99	0.99	1.00	1.01	1.00	0.99	0.98	0.99	1.00	1.01
**Ti**	0.00	0.00	0.00	0.00	0.00	0.00	0.00	0.00	0.00	0.00	0.00	0.00	0.00	0.00	0.00	0.00	0.00	0.00	0.00	0.00	0.00	0.00	0.00
**Fe**	0.00	0.00	0.00	0.00	0.00	0.00	0.00	0.00	0.00	0.00	0.00	0.00	0.00	0.00	0.00	0.00	0.00	0.00	0.00	0.00	0.00	0.00	0.00
**Mn**	0.00	0.00	0.00	0.00	0.00	0.00	0.00	0.00	0.00	0.00	0.00	0.00	0.00	0.00	0.00	0.00	0.00	0.00	0.00	0.00	0.00	0.00	0.00
**Mg**	0.00	0.00	0.00	0.00	0.00	0.00	0.00	0.00	0.00	0.00	0.00	0.00	0.00	0.00	0.00	0.00	0.00	0.00	0.00	0.00	0.00	0.00	0.00
**Ca**	0.00	0.00	0.00	0.00	0.00	0.00	0.00	0.00	0.00	0.00	0.00	0.00	0.00	0.00	0.02	0.00	0.00	0.00	0.00	0.00	0.00	0.00	0.00
**Na**	0.06	0.06	0.02	0.06	0.05	0.03	0.03	0.03	0.03	0.06	0.04	0.04	0.04	0.03	0.03	0.04	0.03	0.03	0.04	0.04	0.03	0.07	0.04
**K**	0.94	0.94	0.96	0.94	0.95	0.95	0.96	0.96	0.96	0.93	0.95	0.97	0.96	0.96	0.95	0.95	0.97	0.96	0.96	0.95	0.96	0.93	0.96
**Total**	5.00	5.00	4.99	5.00	5.00	4.99	5.00	5.00	4.99	4.99	4.99	5.00	4.99	4.99	4.99	4.99	5.00	5.00	5.00	4.99	4.99	5.00	5.00
**End members**																							
**Or**	94.12	94.11	97.55	94.28	95.12	96.99	96.51	96.43	97.00	93.92	95.51	96.40	96.16	96.64	94.83	96.03	96.69	96.47	96.27	95.88	96.45	93.00	96.41
**Ab**	5.83	5.84	2.35	5.67	4.83	2.81	3.44	3.42	2.90	5.98	4.24	3.50	3.79	3.26	3.14	3.82	3.16	3.43	3.68	4.07	3.45	6.90	3.54
**An**	0.05	0.05	0.10	0.05	0.05	0.20	0.05	0.15	0.10	0.10	0.25	0.10	0.05	0.10	2.03	0.15	0.15	0.10	0.05	0.05	0.10	0.10	0.05

**Table 3:** Electron microprobe analyses of albite from tourmaline-bearing leucogranites, sikait, South Eastern desert, egypt.

Mineral	Albite																				
Spot No.	#1	#2	#3	#4	#5	#7	#8	#9	#10	#11	#12	#13	#14	#15	#16	#17	#18	#19	#20	#23	#24
**SiO2**	67.59	69.14	67.17	68.62	67.03	68.01	69.27	66.64	67.99	67.51	69.07	67.23	68.14	67.63	67.57	67.95	68.85	67.45	67.73	68.01	69.12
**TiO2**	0.00	0.01	0.00	0.02	0.00	0.00	0.02	0.00	0.01	0.00	0.00	0.00	0.02	0.01	0.00	0.00	0.06	0.01	0.00	0.00	0.00
**Al2O3**	19.69	20.08	20.15	20.06	19.55	19.86	19.97	19.73	19.82	19.30	19.95	19.17	19.62	20.09	19.95	19.83	19.98	19.91	19.63	19.74	20.15
FeO*	0.00	0.05	0.00	0.12	0.00	0.00	0.04	0.03	0.03	0.00	0.12	0.00	0.19	0.00	0.00	0.00	0.06	0.00	0.00	0.08	0.02
**MnO**	0.00	0.02	0.00	0.01	0.00	0.00	0.01	0.00	0.00	0.00	0.00	0.00	0.11	0.00	0.00	0.00	0.02	0.00	0.00	0.00	0.00
**MgO**	0.00	0.06	0.08	0.00	0.00	0.00	0.02	0.00	0.00	0.04	0.01	0.13	0.04	0.00	0.00	0.00	0.00	0.00	0.00	0.00	0.03
**CaO**	0.38	0.26	1.05	0.41	0.33	0.61	0.42	0.59	0.46	0.83	0.29	0.49	0.22	0.87	0.62	0.51	0.27	0.55	0.37	0.44	0.39
**Na2O**	11.67	10.97	11.13	11.19	11.87	11.56	11.10	11.67	11.52	11.74	11.19	11.29	11.44	11.44	11.65	11.75	11.45	11.78	11.63	11.74	11.34
**K2O**	0.14	0.08	0.21	0.13	0.15	0.13	0.09	0.16	0.14	0.17	0.19	0.20	0.15	0.14	0.11	0.08	0.12	0.11	0.18	0.14	0.19
**P2O5**	0.05	0.06	0.11	0.04	0.00	0.16	0.23	0.06	0.03	0.47	0.02	0.03	0.00	0.08	0.14	0.13	0.03	0.19	0.00	0.07	0.08
**Cr2O3**	0.00	0.02	0.00	0.02	0.00	0.02	0.02	0.00	0.00	0.00	0.00	0.07	0.01	0.00	0.00	0.00	0.03	0.02	0.00	0.00	0.02
**NiO**	0.00	0.05	0.00	0.01	0.00	0.00	0.01	0.00	0.00	0.00	0.00	0.00	0.01	0.00	0.00	0.00	0.00	0.00	0.00	0.00	0.02
**SO3**	0.00	0.00	0.00	0.01	0.00	0.00	0.00	0.00	0.00	0.00	0.00	0.00	0.03	0.00	0.00	0.00	0.01	0.00	0.00	0.00	0.00
**F**	0.00	0.01	0.00	0.00	0.00	0.00	0.00	0.01	0.00	0.00	0.00	0.00	0.00	0.00	0.00	0.00	0.00	0.00	0.00	0.00	0.00
**B2O3**	0.00	0.00	0.00	0.00	0.00	0.00	0.00	0.00	0.00	0.00	0.00	0.00	0.00	0.00	0.00	0.01	0.00	0.00	0.00	0.00	0.00
**ZrO2**	0.00	0.00	0.00	0.00	0.00	0.00	0.00	0.00	0.00	0.00	0.00	0.00	0.00	0.00	0.00	0.00	0.00	0.00	0.00	0.00	0.00
**Nb2O5**	0.00	0.00	0.00	0.01	0.00	0.00	0.00	0.00	0.00	0.00	0.01	0.00	0.01	0.00	0.00	0.00	0.00	0.00	0.01	0.00	0.04
**Ta2O5**	0.00	0.00	0.00	0.00	0.00	0.00	0.00	0.00	0.00	0.00	0.00	0.00	0.05	0.00	0.00	0.00	0.01	0.00	0.00	0.00	0.00
**SnO2**	0.00	0.00	0.01	0.00	0.00	0.00	0.01	0.00	0.00	0.00	0.00	0.00	0.00	0.00	0.00	0.00	0.00	0.00	0.00	0.01	0.00
**HfO2**	0.00	0.00	0.00	0.00	0.00	0.00	0.00	0.00	0.00	0.00	0.00	0.00	0.00	0.00	0.00	0.00	0.00	0.00	0.00	0.00	0.00
**ThO2**	0.00	0.00	0.00	0.00	0.00	0.00	0.00	0.00	0.00	0.00	0.00	0.00	0.00	0.00	0.00	0.00	0.00	0.00	0.00	0.00	101.40
**Total**	99.52	100.81	99.91	100.65	98.93	100.35	101.21	98.89	100.00	100.06	100.85	98.61	100.04	100.26	100.04	100.26	100.89	100.02	99.55	100.23	
	**Cations on the basis of 8 oxygen**																				
**Si**	2.97	2.99	2.95	2.98	2.97	2.97	2.99	2.96	2.98	2.97	2.99	2.99	2.98	2.96	2.96	2.97	2.98	2.96	2.98	2.97	2.98
**Al**	1.02	1.02	1.04	1.03	1.02	1.02	1.02	1.03	1.02	1.00	1.02	1.00	1.01	1.04	1.03	1.02	1.02	1.03	1.02	1.02	1.02
**Ti**	0.00	0.00	0.00	0.00	0.00	0.00	0.00	0.00	0.00	0.00	0.00	0.00	0.00	0.00	0.00	0.00	0.00	0.00	0.00	0.00	0.00
**Fe**	0.00	0.00	0.00	0.00	0.00	0.00	0.00	0.00	0.00	0.00	0.00	0.00	0.01	0.00	0.00	0.00	0.00	0.00	0.00	0.00	0.00
**Mn**	0.00	0.00	0.00	0.00	0.00	0.00	0.00	0.00	0.00	0.00	0.00	0.00	0.00	0.00	0.00	0.00	0.00	0.00	0.00	0.00	0.00
**Mg**	0.00	0.00	0.01	0.00	0.00	0.00	0.00	0.00	0.00	0.00	0.00	0.01	0.00	0.00	0.00	0.00	0.00	0.00	0.00	0.00	0.00
**Zn**	0.00	0.00	0.00	0.00	0.00	0.00	0.00	0.00	0.00	0.00	0.00	0.00	0.00	0.00	0.00	0.00	0.00	0.00	0.00	0.00	0.00
**Ca**	0.02	0.01	0.05	0.02	0.02	0.03	0.02	0.03	0.02	0.04	0.01	0.02	0.01	0.04	0.03	0.02	0.01	0.03	0.02	0.02	0.02
**Na**	1.00	0.92	0.95	0.94	1.02	0.98	0.93	1.00	0.98	1.00	0.94	0.97	0.97	0.97	0.99	1.00	0.96	1.00	0.99	1.00	0.95
**K**	0.01	0.00	0.01	0.01	0.01	0.01	0.00	0.01	0.01	0.01	0.01	0.01	0.01	0.01	0.01	0.00	0.01	0.01	0.01	0.01	0.01
**Total**	5.02	4.96	5.01	4.98	5.03	5.01	4.97	5.03	5.01	5.03	4.98	5.00	5.00	5.01	5.02	5.02	4.99	5.03	5.01	5.02	4.99
	**End members**																				
**Or**	0.77	0.47	1.17	0.74	0.81	0.71	0.52	0.87	0.78	0.91	1.09	1.13	0.85	0.77	0.60	0.44	0.68	0.60	0.99	0.76	1.07
**Ab**	97.48	98.24	93.94	97.29	97.69	96.47	97.44	96.44	97.08	95.37	97.51	96.56	98.11	95.23	96.56	97.23	98.05	96.90	97.30	97.22	97.08
**An**	1.75	1.29	4.90	1.97	1.50	2.81	2.04	2.69	2.14	3.73	1.40	2.32	1.04	4.00	2.84	2.33	1.28	2.50	1.71	2.01	1.85

### Muscovite

The detected white mica (muscovite) is analyzed by EPMA (15 spots) and its structural formulae has been computed (Table 4). The chemical analysis of muscovite grains reveal that they have Al_2_O_3_ (21–25 wt%), FeO* (11–13 wt%), and K_2_O (9–10 wt%). They are primary based up on^50^ diagram (Fig. 6b). The low content of TiO_2_ (av. 0.05 wt%), reflecting the post-magmatic source^51^ (Fig. 6c).

**Table 4:** Electron microprobe analyses of Muscovite from tourmaline- bearing leucogranites, sikait, South Eastern desert, egypt.

Mineral	Muscovite															
Spot No.	#1	#2	#3	#4	#5	#6	#7	#8	#9	#10	#11	#12	#13	#14	#15	
**SiO2**	43.66	41.62	44.29	42.93	42.72	41.42	43.34	42.00	42.73	42.08	42.85	43.59	43.96	43.72	43.19	
**TiO2**	0.05	0.03	0.08	0.02	0.13	0.01	0.00	0.13	0.03	0.00	0.02	0.18	0.03	0.00	0.02	
**Al2O3**	24.27	22.21	24.54	21.39	25.13	22.09	22.93	22.31	21.85	21.88	21.11	23.27	21.72	21.83	21.48	
FeO*	13.16	13.14	11.96	12.22	12.83	13.34	10.94	13.04	11.99	12.46	12.11	11.91	11.45	11.78	11.97	
**MnO**	0.09	3.32	0.07	3.69	0.09	3.84	3.29	0.13	3.53	3.48	3.77	0.12	3.51	3.52	3.64	
**MgO**	0.46	0.08	0.35	0.06	0.22	0.08	0.21	0.59	0.12	0.07	0.07	0.52	0.13	0.09	0.09	
**CaO**	0.02	0.03	0.08	0.03	0.02	0.02	0.06	0.05	0.03	0.01	0.02	0.04	0.05	0.05	0.04	
**Na2O**	0.51	0.09	0.47	0.09	0.36	0.04	0.07	0.52	0.06	0.06	0.06	0.45	0.13	0.04	0.03	
**K2O**	9.67	9.68	9.48	9.93	9.45	9.73	9.28	10.44	9.68	9.87	9.93	9.32	9.63	9.82	9.73	
**P2O5**	0.00	0.00	0.02	0.00	0.00	0.00	0.00	0.00	0.00	0.00	0.00	0.01	0.00	0.00	0.00	
**SO3**	0.00	0.00	0.00	0.00	0.00	0.00	0.00	0.00	0.00	0.00	0.00	0.00	0.00	0.00	0.00	
**F**	5.54	6.18	4.59	6.03	6.17	6.24	5.81	5.84	6.29	6.83	6.55	6.41	5.51	6.45	6.02	
**B2O3**	0.00	0.00	0.00	0.00	0.00	0.00	0.00	0.00	0.00	0.00	0.00	0.00	0.00	0.00	0.00	
**ZrO2**	0.00	0.00	0.00	0.00	0.01	0.00	0.00	0.00	0.00	0.00	0.00	0.01	0.00	0.00	0.00	
**Nb2O5**	0.00	0.01	0.00	0.00	0.00	0.00	0.00	0.00	0.00	0.00	0.00	0.00	0.00	0.00	0.00	
**Ta2O5**	0.00	0.00	0.00	0.00	0.00	0.00	0.00	0.01	0.00	0.00	0.00	0.01	0.00	0.00	0.00	
**SnO2**	0.00	0.00	0.00	0.00	0.01	0.00	0.00	0.00	0.00	0.00	0.00	0.00	0.00	0.00	0.00	
**HfO2**	0.01	0.00	0.00	0.00	0.00	0.00	0.00	0.00	0.00	0.00	0.00	0.00	0.00	0.00	0.00	
**ThO2**	0.00	0.00	0.00	0.00	0.00	0.00	0.00	0.00	0.00	0.00	0.00	0.00	0.00	0.00	0.00	
**Total**	97.44	96.39	95.93	96.39	97.14	96.81	95.93	95.06	96.31	96.74	96.49	95.85	96.12	97.30	96.21	
	**Cations on the basis of 22 oxygen**															
**Si**	6.32	6.25	6.41	6.42	6.21	6.22	6.40	6.32	6.38	6.31	6.42	6.41	6.50	6.44	6.44	
**Al iv**	1.68	1.75	1.59	1.58	1.79	1.78	1.60	1.68	1.62	1.69	1.58	1.59	1.50	1.56	1.56	
**Al vi**	2.45	2.18	2.60	2.18	2.52	2.13	2.39	2.27	2.22	2.17	2.15	2.44	2.29	2.24	2.22	
**Ti**	0.01	0.00	0.01	0.00	0.01	0.00	0.00	0.01	0.00	0.00	0.00	0.02	0.00	0.00	0.00	
**Cr**	0.00	0.00	0.00	0.00	0.00	0.00	0.00	0.00	0.00	0.00	0.00	0.00	0.00	0.00	0.00	
**Fe**	1.59	1.65	1.45	1.53	1.56	1.68	1.35	1.64	1.50	1.56	1.52	1.46	1.42	1.45	1.49	
**Mn**	0.01	0.42	0.01	0.47	0.01	0.49	0.41	0.02	0.45	0.44	0.48	0.01	0.44	0.44	0.46	
**Mg**	0.10	0.02	0.08	0.01	0.05	0.02	0.05	0.13	0.03	0.02	0.02	0.11	0.03	0.02	0.02	
Li*	0.96	1.12	0.79	1.08	1.08	1.13	1.03	1.05	1.13	1.24	1.18	1.13	0.98	1.15	1.08	
**Ca**	0.00	0.00	0.01	0.00	0.00	0.00	0.01	0.01	0.00	0.00	0.00	0.01	0.01	0.01	0.01	
**Na**	0.14	0.03	0.13	0.03	0.10	0.01	0.02	0.15	0.02	0.02	0.02	0.13	0.04	0.01	0.01	
**K**	1.78	1.85	1.75	1.89	1.75	1.86	1.75	2.00	1.84	1.89	1.90	1.75	1.82	1.85	1.85	
**Fe/Fe + Mg**	0.94	0.99	0.95	0.99	0.97	0.99	0.97	0.93	0.98	0.99	0.99	0.93	0.98	0.99	0.99	
**Mn/Mn + Fe**	0.01	0.20	0.01	0.23	0.01	0.23	0.23	0.01	0.23	0.22	0.24	0.01	0.24	0.23	0.24	

### Tourmaline

The well-developed crystals of tourmaline crystals have been analyzed by using EPMA (20 spots) and their structural formulae have been calculated based up on 31 anions. In addition, H_2_O and B_2_O_3_ were estimated based on of F + OH = 4 apfu, while B = 3 apfu^52^. The examined tourmaline has FeO* varies from 11.89 to 16.52 wt%, MgO from 0.99 to 2.03 wt%, Na_2_O from 2.01 to 2.11 wt%, B_2_O_3_* from 10.10 to 10.46 wt%, and H_2_O* from 3.11 to 3.47 wt%. It is noticeable that the examined tourmaline has a high Si and Al (5.89 and 6 apfu, respectively) contents (Table 5). It is noticeable that there is a slight difference in SiO_2_ (av. 30.05 wt%), Al_2_O_3_ (av. 32. 57 wt%), Na_2_O (av. 2.05 wt%), K_2_O (av. 0.04 wt%), H_2_O*(av. 3.34 wt%), and B_2_O_3_*(av. 10.28 wt%) from the examined tourmaline enclosed in leucogranites relative to those enclosed in schist (metapelite)^3^. However, the main difference is corresponding to their FeO* (av. 13.66 wt% for the examined study and 4.92 wt% for those enclose in schist) and MgO (av. 1.56 wt% for the examined study and 8.85 wt% for those enclose in schist) contents, which may be related to diffusion of Fe and Mg enrichment during metamorphism. Notably, the analyzed tourmaline has high contents of Si (reaches up to 5.92 apfu) and Al (reaches up to 6 apfu, which occupy the Z-site and the surplus occupies Y-site). The analyzed tourmaline is ascribed to alkali group^52^ (Fig. 6d). In the Mg-Ca-Fe and Fe-Al-Mg ternary diagrams^53^ the examined tourmaline samples plot in the field of Li-poor granitoid (Figs. 6e-f).

**Table 5:** Electron microprobe analyses of tourmaline from tourmaline- bearing leucogranites, sikait, South Eastern desert, egypt.

Spot No.	#1	#2	#3	#4	#5	#6	#7	#8	#9	#10	#11	#12	#13	#14	#15	#16	#17	#18	#19	#20
**SiO2**	35.42	35.16	35.48	35.22	34.69	35.73	35.46	35.09	35.44	33.39	35.22	35.12	35.35	34.43	35.18	34.13	35.38	35.53	34.92	34.72
**TiO2**	0.14	0.14	0.11	0.11	0.17	0.18	0.18	0.18	0.13	0.15	0.19	0.22	0.18	0.16	0.13	0.15	0.22	0.21	0.17	0.19
**Al2O3**	29.23	31.06	32.57	32.97	32.13	32.48	32.81	33.37	32.74	32.22	33.27	33.04	33.03	33.04	33.07	33.06	32.71	32.83	32.46	33.27
**Cr2O3**	0.02	0.02	0.04	0.04	0.04	0.02	0.02	0.06	0.01	0.03	0.01	0.05	0.06	0.02	0.01	0.03	0.05	0.01	0.02	0.02
FeO*	15.74	14.24	12.41	12.41	14.17	15.18	13.68	11.94	13.96	16.52	11.96	14.23	11.94	14.04	12.96	14.02	15.73	11.89	13.17	12.96
**MgO**	1.65	1.65	1.51	1.51	1.76	1.45	1.45	1.26	1.62	1.53	1.75	1.39	0.99	1.54	1.62	1.53	1.39	2.03	1.76	1.75
**CaO**	0.64	0.38	0.15	0.15	0.13	0.54	0.33	0.27	0.17	0.26	0.32	0.25	0.38	0.18	0.17	0.26	0.34	0.46	0.13	0.17
**MnO**	0.38	0.39	0.52	0.52	0.27	0.47	0.47	0.52	0.49	0.55	0.46	0.43	0.52	0.53	0.49	0.55	0.43	0.39	0.33	0.46
**NiO**	0.00	0.00	0.00	0.00	0.00	0.00	0.00	0.00	0.00	0.00	0.00	0.00	n.d.	0.00	0.00	0.00	0.00	0.00	0.00	0.00
**Na2O**	2.11	2.11	2.02	2.02	2.07	2.03	2.03	2.01	2.04	2.03	2.06	2.04	2.01	2.04	2.04	2.03	2.04	2.07	2.07	2.06
**K2O**	0.05	0.05	0.04	0.04	0.05	0.04	0.04	0.04	0.04	0.04	0.04	0.05	0.04	0.06	0.04	0.04	0.05	0.04	0.05	0.04
**P2O5**	0.02	0.02	0.01	0.01	0.04	0.00	0.00	0.00	0.04	0.07	0.02	0.03	0.00	0.01	0.04	0.07	0.03	0.02	0.04	0.01
**SO3**	0.00	0.00	0.00	0.00	0.00	0.00	0.00	0.00	0.00	0.00	0.00	0.00	0.00	0.00	0.00	0.00	0.00	0.00	0.00	0.00
**F**	0.78	0.78	0.32	0.35	0.45	0.79	0.67	0.34	0.29	0.45	0.40	0.35	0.31	0.29	0.31	0.43	0.27	0.44	0.48	0.33
H2O*	3.11	3.14	3.39	3.38	3.30	3.23	3.26	3.39	3.44	3.28	3.38	3.41	3.40	3.40	3.41	3.32	3.47	3.37	3.30	3.39
B2O3*	10.10	10.16	10.27	10.28	10.19	10.46	10.37	10.29	10.36	10.13	10.34	10.36	10.28	10.25	10.32	10.21	10.44	10.37	10.22	10.29
Li2O*	0.44	0.30	0.40	0.36	0.13	0.21	0.30	0.47	0.19	0.00	0.41	0.21	0.58	0.11	0.28	0.10	0.08	0.45	0.24	0.22
**Total**	96.29	96.67	96.26	95.66	96.91	98.69	97.37	95.44	97.52	96.79	96.26	97.63	95.67	96.84	96.95	96.23	99.40	96.64	96.10	96.18
	**Cations on the basis of 31 oxygen**																			
**T: Si**	6.10	6.01	6.00	5.96	5.92	5.94	5.94	5.93	5.95	5.73	5.92	5.89	5.98	5.84	5.93	5.81	5.89	5.96	5.94	5.86
**Al**	0.00	0.00	0.00	0.04	0.08	0.06	0.06	0.07	0.05	0.27	0.08	0.11	0.02	0.16	0.07	0.19	0.11	0.04	0.06	0.14
**B**	3.00	3.00	3.00	3.00	3.00	3.00	3.00	3.00	3.00	3.00	3.00	3.00	3.00	3.00	3.00	3.00	3.00	3.00	3.00	3.00
**Z: Al**	5.93	6.00	6.00	6.00	6.00	6.00	6.00	6.00	6.00	6.00	6.00	6.00	6.00	6.00	6.00	6.00	6.00	6.00	6.00	6.00
**Mg**	0.07	0.00	0.00	0.00	0.00	0.00	0.00	0.00	0.00	0.00	0.00	0.00	0.00	0.00	0.00	0.00	0.00	0.00	0.00	0.00
**Cr**	0.00	0.00	0.00	0.00	0.00	0.00	0.00	0.00	0.00	0.00	0.00	0.00	0.00	0.00	0.00	0.00	0.00	0.00	0.00	0.00
**Fe3+**	0.00	0.00	0.00	0.00	0.00	0.00	0.00	0.00	0.00	0.00	0.00	0.00	0.00	0.00	0.00	0.00	0.00	0.00	0.00	0.00
**Y: Al**	0.00	0.26	0.50	0.53	0.38	0.30	0.42	0.57	0.42	0.24	0.51	0.42	0.56	0.44	0.49	0.44	0.31	0.44	0.44	0.49
**Ti**	0.02	0.02	0.01	0.01	0.02	0.02	0.02	0.02	0.02	0.02	0.02	0.03	0.02	0.02	0.02	0.02	0.03	0.03	0.02	0.02
**V**	0.00	0.00	0.00	0.00	0.00	0.00	0.00	0.00	0.00	0.00	0.00	0.00	0.00	0.00	0.00	0.00	0.00	0.00	0.00	0.00
**Cr**	0.00	0.00	0.01	0.01	0.01	0.00	0.00	0.01	0.00	0.00	0.00	0.01	0.01	0.00	0.00	0.00	0.01	0.00	0.00	0.00
**Fe3+**	0.00	0.00	0.00	0.00	0.00	0.00	0.00	0.00	0.00	0.00	0.00	0.00	0.00	0.00	0.00	0.00	0.00	0.00	0.00	0.00
**Mg**	0.35	0.42	0.38	0.38	0.45	0.36	0.36	0.32	0.41	0.39	0.44	0.35	0.25	0.39	0.41	0.39	0.35	0.51	0.45	0.44
**Mn**	0.06	0.06	0.07	0.07	0.04	0.07	0.07	0.07	0.07	0.08	0.07	0.06	0.07	0.08	0.07	0.08	0.06	0.06	0.05	0.07
**Fe2+**	2.27	2.04	1.76	1.76	2.02	2.11	1.92	1.69	1.96	2.37	1.68	2.00	1.69	1.99	1.83	2.00	2.19	1.67	1.87	1.83
**Zn**	0.00	0.00	0.00	0.00	0.00	0.00	0.00	0.00	0.00	0.00	0.00	0.00	0.00	0.00	0.00	0.00	0.00	0.00	0.00	0.00
Li*	0.31	0.20	0.27	0.24	0.09	0.14	0.20	0.32	0.13	0.00	0.28	0.14	0.40	0.08	0.19	0.07	0.06	0.30	0.17	0.15
**∑Y**	3.00	3.00	3.00	3.00	3.00	3.00	3.00	3.00	3.00	3.10	3.00	3.00	3.00	3.00	3.00	3.00	3.00	3.00	3.00	3.00
**X: Ca**	0.12	0.07	0.03	0.03	0.02	0.10	0.06	0.05	0.03	0.05	0.06	0.04	0.07	0.03	0.03	0.05	0.06	0.08	0.02	0.03
**Ba**	0.00	0.00	0.00	0.00	0.00	0.00	0.00	0.00	0.00	0.00	0.00	0.00	0.00	0.00	0.00	0.00	0.00	0.00	0.00	0.00
**Na**	0.70	0.70	0.66	0.66	0.68	0.65	0.66	0.66	0.66	0.68	0.67	0.66	0.66	0.67	0.67	0.67	0.66	0.67	0.68	0.67
**K**	0.01	0.01	0.01	0.01	0.01	0.01	0.01	0.01	0.01	0.01	0.01	0.01	0.01	0.01	0.01	0.01	0.01	0.01	0.01	0.01
**Rb**	0.00	0.00	0.00	0.00	0.00	0.00	0.00	0.00	0.00	0.00	0.00	0.00	0.00	0.00	0.00	0.00	0.00	0.00	0.00	0.00
**Cs**	0.00	0.00	0.00	0.00	0.00	0.00	0.00	0.00	0.00	0.00	0.00	0.00	0.00	0.00	0.00	0.00	0.00	0.00	0.00	0.00
**Vacancies**	0.17	0.22	0.30	0.30	0.28	0.24	0.27	0.28	0.30	0.27	0.26	0.28	0.26	0.28	0.29	0.27	0.27	0.24	0.28	0.29
**OH**	3.58	3.58	3.83	3.81	3.76	3.58	3.64	3.82	3.85	3.76	3.79	3.81	3.83	3.84	3.83	3.77	3.86	3.77	3.74	3.82

**Fig. 7:**
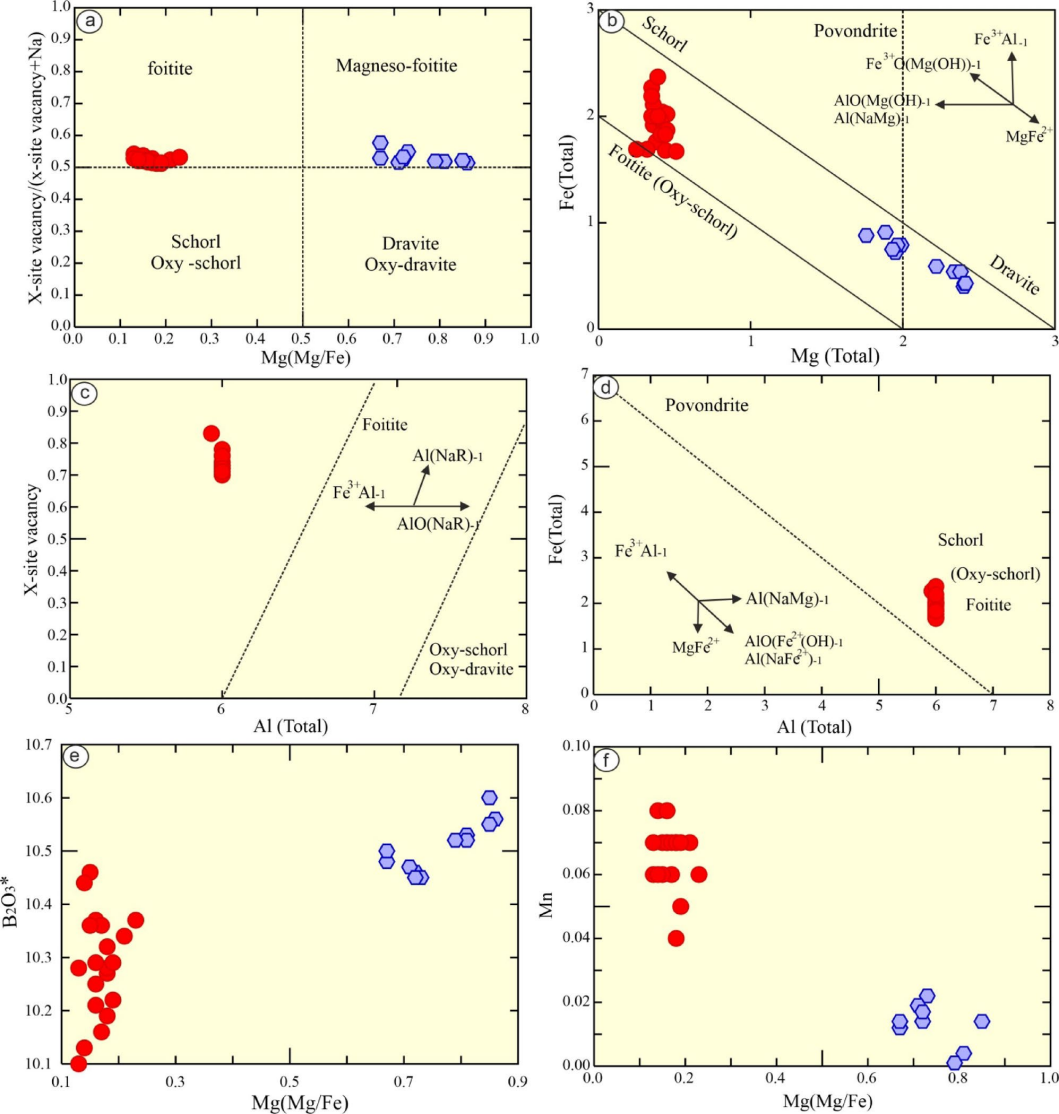
Tourmaline chemistry diagrams: (**a**) Mg/(Fe + Mg) -X-site vacancy/(Na + X-site vacancy)^53^; (**b**) Fe^Total^- Mg^Total^; **c**) X-site vacancy-Al^Total^; (**d**) Fe^Total^-Mg^Total^; (**e**) Mg# - B_2_O_3_*; and (**f**) Mg# - Mn diagrams.

They are of foitite end-member, whereas those enclosed in schist (metapelite) are of dravite end- members according to^53^ diagram (Figs. 7a-c). Notably, the examined tourmaline are controlled by Fe^3+^ substitution based up on the vector of Fe^3+^Al_−1_ (Fig. 7b), in comparison with Fe^2+^Al_−1_ for those enclosed in metapelite. The tourmaline compositions was controlled by ❑Al(NaR)_−1_ substitution vector as indicated in X-site vacancy-Al^Total^ diagram^53^ (Fig. 7c). In Fig. 7d (Al^Total^-Fe^Total^), tourmaline follow the MgFe^2+^_−1_, reflecting Mg, Al, and Na variations. On the other hand, the examined tourmaline contain low contents of Na# and Mg# (Figs. 7e-f), which varies from 0.86 to 0.97 and 0.13 to 0.23, respectively. The low Na contents of the examined tourmaline (avg. 0.67 apfu), suggesting that they were precipitated from low/moderate salinity fluids^6^.

### Zircon

Structural formulae and composition of the examined zircon crystals are in supplementary Table S1. A few spot analysis (five) of zircons reveal that they have SiO_2_ varies from 31.32 to 32.08 wt%, ZrO_2_ from 63.09 to 63.56 wt%, and with appreciable HfO_2_ (av. 1.66 wt%) content.

### Non-silicate minerals

Apatite has been analyzed (11 spots) and it is structural formulae has been computed (Supplementary table S2). It is composed mainly of CaO (av. 52.83 wt%), and P_2_O_5_ (34.64),with appreciable contents of FeO* (2.03 wt%), F (3.65 wt%) and SO_3_ (3.49 wt%). On the other hand, the analyzed magnetite (7 spots) has appreciable content of FeO* ranges from 87.6 to 92.56 wt%, with less amount of TiO_2_ (av. 0.77 wt%) and MgO (av. 00.01 wt%) (Supplementary table S3). Eleven spots analysis of goethite minerals reveal that they are have FeO* ranges from 43.16 to 58.87 wt%, SiO_2_ from 4.99 to 6.72 wt%, and CaO (0.94–2.64 wt%) (Supplementary table S4).

## Discussion

### REEs tetrad effect

The abundance of distinct REEs in the crust demonstrates significant variance. Generally, their abundance declines with increases in their atomic number, as per the Oddo-Harkins rule which predicts that elements with even atomic numbers are more numerous than those with odd atomic numbers^55^. Thus, Ce is the highest REEs abundant in the crust, whereas the Lu is much rare. However, the last one still higher some of precise metals like gold and silver. They are commonly present as trivalent cations. The ionic radii of REE ions decrease as the atomic number rises. This phenomenon called lanthanide contraction^56^. HREEs are generally found as oxides or phosphate minerals with 6 to 8 coordination, whereas LREEs are frequently found as carbonates and phosphates with 8 to 10 coordination. Silicate minerals can also include both LREEs and HREEs^55,57^. In the crust, the LREEs are much high abundant than HREEs. Some REEs-bearing minerals can increase their abundance. For example, monazite represent the primary LREE-bearing minerals, whereas apatite and xenotime increase the HREEs abundance^49,58^. Hydrothermal fluids (Post-magmatic metasomatism) and magmatic evolution, usually affect the activation and transport of REEs^55,57^.

Herein, the REEs tetrad effect are investigated through two famous methods. These methods are the oldest^42^ and the youngest lambda method^46^. Some authors^59,60^ subdivided the fourteen lanthanide elements into four groups (T1, T2, T3, and T4). The second tetrad is difficult to recognize it as a result of negative behavior of Eu and naturally absence of Pm occurrence^46^. REEs pattern may reveal W-type and/or M-type^60^. The REE tetrad effect is most visible in late magmatic differentiates especially with strong hydrothermal interactions or deuteric alteration. Moreover, the tetrad effect is often accompanied by geochemical behavior of many isovalent traces (Sect. 5.3)^49,58^. According to recent arguments, peraluminous magmatic systems are the result of the transition from a silicate melt to a high-temperature hydrothermal regime. As a result, chemical complexation with a range of ligands is primarily responsible for controlling the geochemical behavior of the isovelant incompatible elements in highly evolved granitic rocks^58,61^. Through visual inspection, the examined samples have M-type tetrad in T1 (av. 1.06) tetrad (and lack of W-type), and their intensities (T1,3) range from 1.04 to 1.37.

On the other hand, and to avoid Irber^42^ method problems such as measuring of all 4 elements that don’t eliminate the main problem of T2; arbitrary Ce and Eu (as they have two oxidation states) anomalies, we further used lambda method^46^. Therefore, τ1, τ2, τ3, and τ4 are tetrad coefficients resulted from^46^. Each tetrad coefficients may be has positive or negative values, reflecting M-type or W-type, respectively. Coefficients tetrad values of τ1 (0.68–1.42), τ2 (0.05–0.6), τ3 (0.095–0.63) and τ4 (0.14–0.56), suggesting M-type tetrad. It is noticeable that the obtained results certainly in the first segment from Irber^42^ are closely similar to those of^46^. Further constraints, true Ce and Eu anomalies can be obtained by lambda methods, where Eu (av. 0.026) and Ce (av. 1.1) anomalies, which are very close to those obtained from ^42^(av. 0.02, 1.04, respectively).

### Magma type

**Fig. 8:**
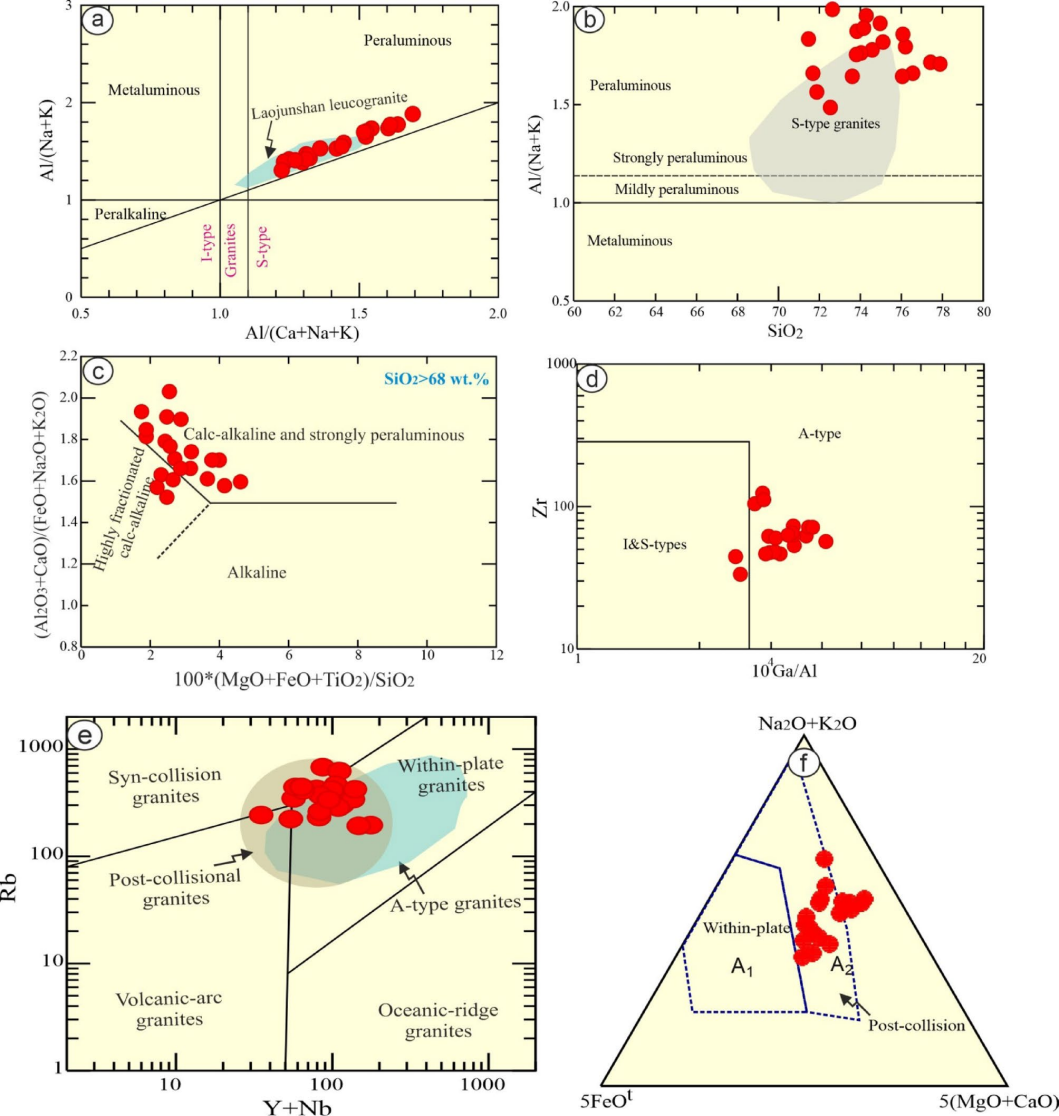
Bulk rock geochemical diagrams: (**a**) A/NK - A/CNK of^62^. Green field is of suite of Laojunshan S-type leucogranites, southwest China^64^; (**b**) SiO_2_ - A/CNK diagram. Grey field is of S-type granites after^38^; (**c**) Binary diagram of major oxides with (SiO_2_ > 68%)^67^;(**d**) Ga/Al-Zr^70^; (**e**) Rb- Nb + Y^68^; and **f**) 5*FeO^t^- Na_2_O + K_2_O- 5(MgO + CaO) ternary diagram^69^.

The investigated samples have A/CNK^62^ >1.1, reflecting strongly peraluminous S-type (as indicated by the presence of muscovite)^63^, which are similar to Egyptian Abu Had leucogranites^38^ and S-type leucogranites of Laojunshan area, southwest China^64^ (Figs. 8a-b). In addition, S-type can be inferred by their high silica SiO_2_ (av. 74 wt%) content and Rb/Zr (av. 6.13)^65,66^.

They are sub-alkaline as indicated by TAS (Fig. 5a) diagram^32^. Their calc-alkaline is indicated by agpaitic index (AI), which is lower than 0.87 (0.55–0.79). They range from highly evolved rocks to calc-alkaline^67^ (Fig. 8c). Additionally, their SiO_2_ values and Fe*(Fe/Fe + Mg) (0.79–0.95), suggesting a ferroan signature^35^. Granitic rocks with geochemical composition of highly fractionated S-and I- types (particularly, strongly peraluminous) are mostly have characteristics of post- collision and A-type granites^65,68–70^.

The investigated rocks share the geochemical features of post- collision and A-type granites (Fig. 8d), which they exhibit a notable enrichment of LFSEs in compared to HFSEs with strong Ba, Sr and Ti negative anomalies (Fig. 5e). Furthermore, Rb/Sr ratio infer the petrogenetic affinity of these rocks^71^. The examined rocks possess Rb/Sr ratio more than unity (10.52–39.34), suggesting anorogenic signature^71^. In addition, high concentrations of SiO_2_, Fe_2_O_3_/MgO, Na_2_O/K_2_O, Nb, Zr, Zn, Ga/Al with depletion of CaO, MgO and Fe_2_O_3_ support the composition of A-type rather than S- and I-type rocks^70,72,73^ (Fig. 8d).

The examined rocks have elements similar to those of the Baijuhuajian granite^43^ and Yangmeiwan granite^44^ suggesting A-type affinity. They have no Nb and Ta anomalies but extensive depletion in Ti, Eu, Ba, P, and Sr. Additionally, they have low La/Nb (av. 0.03) ratio, which is in accord with A-type affinity (La/Nb < 1)^43^. Their Nb/Ta (av. 4.53) ratio also is consistent with A-type granites^43,69,72^. Furthermore, it’s noticeable that the REE patterns of the examined rocks reveal a distinctive character, which is clearly analogous to post-orogenic rather than those of collision ones (Fig. 5f). Therefore, the examined granitic rocks have calc-alkaline, peraluminous of post-collision affinity. Accordingly, the examined rocks are of A-type affinity^70^ (Fig. 8d-f). According to Y + Nb-Rb binary discrimination diagram^68^ the studied rocks overlapping A-type granites, within plate, and post-collision granites (Fig. 8e). The A-type possesses A1 of within plate mantle nature and A2 of crustal and post-collisional granites^69,72^. Accordingly, the investigated leucogranites straddle A2 field^69,72^ (Fig. 8f).

### Sources and origin of leucogranites

**Fig. 9:**
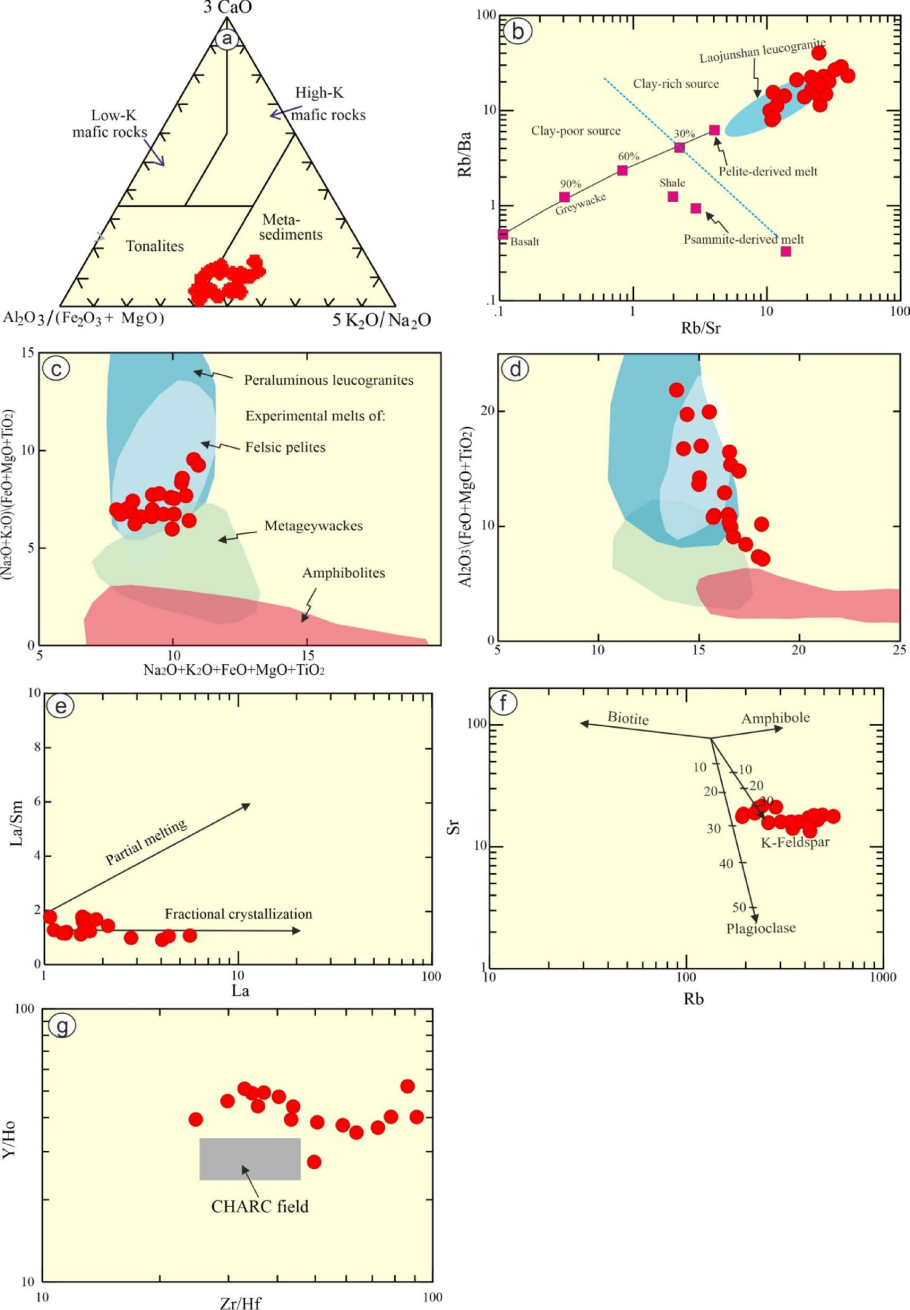
Bulk rock geochemical diagrams: (**a**) Source ternary diagram^89^; (**b**) Rb/Sr-Rb/Ba^64^; **c**,(** d**) Sources discrimnation binary diagram^90^. Fields of greywackes, pelites and amphibolites are from Patiño Douce^91^. Peraluminous leucogranites^38^; (**e)** La/Sm-La^85^; (**f**) Sr vs. Rb diagrams^92^; and (g) Y/Ho vs., Zr/Hf diagram^58^.

Egyptian calc-alkaline granitic rocks are extruded during syn- and late- to post-collisional stages. Some of the latter rocks are either geochemically specialized (not mineralized) or mineralized^74^. The generation of the examined leucogranites may be ascribed to fractionation of mafic rocks (due to large quantities of the surrounded mafic-ultramafic rocks). One granitic body can be produced by fractional crystallization of nine mafic bodies^75^. However, the examined rocks have chemical composition (SiO_2_ > 72, K_2_O + Na_2_O > 7, MgO < 0.2, Cr = 1.09, and Ni = 1.65), reflecting no mafic magma complement, dispute the creation of them by fractional crystallization from mantle-derived rocks. In addition, their high contents of SiO_2_, K_2_O + Na_2_O, and LILEs (Rb), reflecting that they cannot be generated from high melting degree of a granulitic residue. Furthermore, both of the previous hypothesis yield magma of metaluminous to weakly peraluminous with high Sr abundances, which are contrast to the case of study (strongly peraluminous and the average Sr content is 17.13 ppm). Nevertheless, they can be formed by low degree of melting of crustal rocks such as Al-rich metasedimentary (e.g. schist) rocks^76,77^ (Fig. 9a). High concentration of Rb suggesting the examined rocks are related to mica -rich sources (metasedimentary rocks)^39,64,78,79^.

The crustal origin of the examined rocks can be manifested through some ratios such as high Rb/Sr (10.52–39.34, which are greater than the mantle value < 0.1), Rb/Ba (7.35–38.5), and low Sr/Ba (0.45–1.6), and Rb/Zr (1.57–11). High LFSEs/HFSEs ratio such as Rb/Nb, which varies from 1.1 to 10.62, reflecting their derivation from crustal rocks by partial melting^80^. High A/CNK of the studied peraluminous leucogranites may be ascribed to partial melting of metasediments^78,79^. This is supported by low FeO + MgO (< 4 wt%) content^38,81,82^. The pelitic parent for the examined rocks can be inferred through Rb enrichment relative to Sr, and high K_2_O^83^. According to binary diagram of Rb/Sr-Rb/Ba, the studied leucogranites are formed from clay-rich pelitic sources comparable to Laojunshan S-type^64^ (Fig. 9b), as well as peraluminous leucogranites and felsic pelite fields (Figs. 9c-d). In addition, they have high ratio of Al_2_O_3_/TiO_2_ (363–1546), reflecting partial melting of metasediments^66^.

Further constrains, to distinguish between pelite (clay-rich and plagioclase- poor) and psammite (plagioclase- rich), some ratio can be used such as CaO/Na_2_O. Pelite rocks have CaO/Na_2_O less than 0.3, whereas psammite are more than this value (> 0.3)^39^,. The examined leucogranites have low CaO/Na_2_O ranges from 0.08 to 0.15, suggesting their derivation from metapelite-S-type melts.

**Fig. 10:**
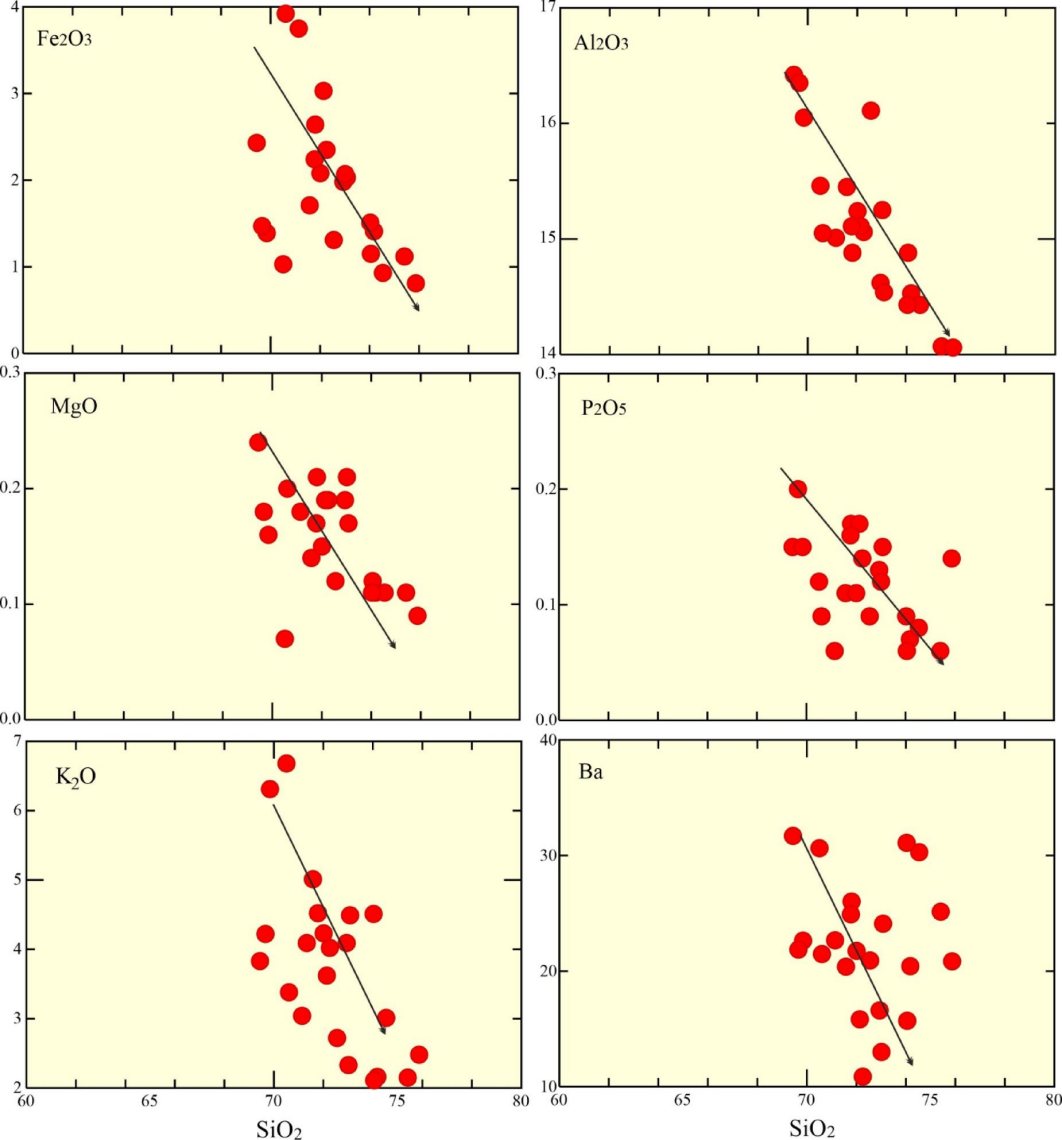
Harker variation diagrams (SiO_2_ – Fe_2_O_3_, Al_2_O_3_, MgO, P_2_O_5_, K_2_O, and Ba).

Alternatively, the rocks studied have low contents of Ba (av. 22.21 ppm), and Sr (av. 17.13 ppm), as well as high Rb (192–679 ppm) concentration (Table 1), suggesting fractional crystallization after crustal melting^84^. In terms of, La-La/Sm binary diagram^85^ reveals the partial melting and fractionation degrees, the examined granitic rocks follow strong fractionation trend (Fig. 9e). The predominant fractionation trend is feldspar (K-feldspar) according to binary diagram Rb-Sr (Fig. 9f). This is supported by (1) strong positive Pb, Rb, and K (trace elements pattern); (2) Sr, Ti, Ba, and Eu anomalies (negative); and (3) negative relationships between SiO_2_ versus Fe_2_O_3_, Al_2_O_3_, MgO, P_2_O_5_, K_2_O and Ba (Fig. 10), reflecting highly fractional crystallization of feldspars, apatite, Fe-Ti oxides fractionally crystallized.

Comparable geochemical activity of isovalents like Hf, Zr, Y, Ho, Nb, La, and Zr can be used to deduce the effect of metasomatism, because they have the same radius and charges (CHARC). Zr/Hf ratio, varies from 24.72 to 488.4 (av. 82.94), is higher than the chondritic limit (33–40^42^. According to Dostal and Chatterjee^61^ these isovalents are stable in magmatic melt but may undergo metasomatic modifications. In addition, non-CHARC values of Y/Ho, which ranges from 27.48 to 54.62 (av. 43.36), higher than the chondritic value, reflecting a metasomatic effect as well as fluorine and boron complexation^58^. The studied samples do not conform to dominantly CHARAC behavior, as shown by a plot of Y/Ho against Zr/Hf^58^ (Fig. 9g). Further constraints, the post-magmatic metasomatism is supported by non-chondritic values of Nb/Ta, La/Nb, which are lower than the chondritic values (av. 4.53; 0.03, and 0.15, respectively)^86^. The non-CHARAC behavior for the examined samples, reflect a physicochemical characteristics of the magma^42^. These magma are commonly enriched by B, H_2_O, and F, which represent a transitional magma varies from pristine melts to post-magmatic metasomatism (hydrothermal fluids)^49,58^. Finally, the studied leucogranites are developed within extension tectonic regime during post-collisional episode (mixed magmatic and post magmatic geologic processes), resulting thinning of lithosphere, crustal delamination and upwelling of asthenosphere, which led to melting of the crustal clay- rich rocks at low temperatures (663–786 °C), depending on saturation temperatures of zircon^87^. The calculated TZr is lower than those of A-type granites in the ANS (750–830 °C)^88^A2 of Homrit Mukpid granites (avg. 784 °C^22^) and A1-Type (rifting-related) felsic rocks of Khan et al.^73^.

### Tourmaline genesis

Tourmaline origin may be attributed to one of the following hypotheses; (1) hypogene boron-rich granitic magma; and (2) Metasomatic (post-magmatic) by B-rich fluids through micro-fractures (fluid-rock interaction between later phase of felsic intrusions and the host rocks)^93–95^. The tourmaline-bearing metapelitic schist, Sikait area, Egypt, is attributed to the interaction between B-bearing pegmatite and the host rocks (metapelite)^3,96^. It’s noticeable that the examined tourmaline nodules are ascribed to magmatic (B-rich granitic magma)^97^. This is indicated by medium- coarse grained and subhedral to euhedral tourmaline crystals as indicated by petrographic examinations, reflecting their magmatic origin. Generally, the tourmalines reflect the host rocks compositions with the high Al^Total^ (5.93–6.00 apfu) and low Ca (0.02–0.12 apfu), Mg (0.25–0.51 apfu), and Fe^Total^ (1.67–2.37 apfu) values. Therefore, it is most likely that the examined tourmaline form directly during the late-magmatic stage. However, the presence of undulose quartz, turbid feldspar (due to sericitization, saussuritization and kaolinitization) and fractured tourmaline crystals grains as well as isovelant indices (HFSEs), reflecting the presence of tourmaline in the magma late stage and the role of post-magmatic metasomatism as indicated by non-CHARC values.

## Conclusions

The current study reports a new field, petrographic, and bulk rock geochemistry of tourmaline-bearing leucogranites. These rocks are calc-alkaline, hyperaluminous with ferroan signature S-type granites. They remarkably high total alkalis, silica, Rb, Ga/Al, Zn, Pb, and LILEs (e.g. Rb) in comparison with HFSEs. They exhibit pronounced Ti, Sr, Eu, and Ba depletions. Their REEs exhibit M-type tetrad (av. t1 = 1.06). Furthermore, they have positive lambda coefficient τ1 - τ4, suggesting M tetrads. These leucogranites formed within extension tectonic regime during post-collisional episode, resulting raising of asthenosphere, which led to melting of the surrounding clay-rich pelite crustal rocks followed by magmatic fractionation and crustal contamination as indicated by Zr/Nb and Ba/Nb ratios. Crustal source involvement for the studied leucogranites can be deduced by using some ratios like high Rb/Nb, Rb/Sr and low Sr/Ba and Rb/Zr. Non-chondritic values of Zr/Hf, Y/Ho, Nb/Ta, La/Nb, reflecting the main role of post-magmatic metasomatism for tourmaline formation. Tourmaline occurs as medium sized grains and subhedral tourmaline crystals with FeO* (11.89–16.52 wt%), MgO (0.99–2.03 wt%), Na_2_O (2.01–2.11 wt%), and B_2_O_3_* (10.10–10.46 wt%), suggesting alkali-group and foitite end-member.

## Electronic supplementary material

Below is the link to the electronic supplementary material.

Supplementary Material 1

## Data Availability

The analyzed data of this work are presented in this manuscript.
